# Serum prolactin and gonadal hormones in hemodialysis women: a meta-analysis

**DOI:** 10.1186/s12902-023-01452-w

**Published:** 2023-09-25

**Authors:** Kailu Zhang, Lanbo Zhao, Yadi Bin, Miao Guo, Xue Zhou, Min Li, Lu Han, Qiling Li

**Affiliations:** Department of Obstetrics and Gynecology, the First Affiliated Hospital of Xi’an Jiaotong University, Xi’an, 710061 China

**Keywords:** Hemodialysis, Sex hormones, Prolactin, Luteinizing hormone, Follicle-stimulating hormone

## Abstract

**Background:**

A meta-analysis followed by PRISMA 2020 statement was performed aiming to present a whole prolactin and sex hormone profile in hemodialysis women.

**Methods:**

Literatures were searched in PubMed, Cochrane library, Embase, and Web of science before March 11, 2023. Trial sequential analysis (TSA) was performed to test the conclusiveness of this meta-analysis. Egger’s test and trim-and-fill analysis was used to test publication bias. We took standardized mean difference (SMD) as pool effect of hormones values including prolactin (PRL), follicle-stimulating hormone (FSH), luteinizing hormone (LH), estradiol (E_2_) and progesterone (P). This study was registered in PROSPERO and the number was CRD42023394503.

**Results:**

Twenty-two articles from 13 countries were analyzed. Combining the results of TSA and meta-analysis, we found that compared with healthy control, hemodialysis women had higher PRL, follicular FSH and LH values and lower P levels (PRL: *I*^*2*^ = 87%, SMD 1.24, 95% CI: 0.79–1.69, *p* < 0.00001; FSH: *I*^*2*^ = 0%, SMD 0.34, 95% CI: 0.13–0.55, *p* = 0.002; LH: *I*^*2*^ = 39%, SMD 0.64, 95% CI: 0.34–0.93, *p* < 0.00001; P: *I*^*2*^ = 30%, SMD − 1.62, 95% CI: -2.04 to -1.20, *p* < 0.00001). What’s more, compared with women after renal transplantation, hemodialysis women had higher PRL levels (*I*^*2*^ = 0%, SMD 0.51, 95% CI: 0.25–0.78, *p* = 0.0001). There was not enough evidence to draw a conclusion on the comparison of hormones between regular and irregular menses hemodialysis women. Egger’s test and trim-and-fill analysis didn’t show significant publication bias.

**Conclusions:**

Hemodialysis women had higher serum PRL, follicular phase FSH, LH and lower serum P values compared with healthy control. PRL values of hemodialysis women were also higher than that of women after renal transplantation.

**Supplementary Information:**

The online version contains supplementary material available at 10.1186/s12902-023-01452-w.

## Introduction

Women with chronic kidney disease, especially end-stage renal disease, usually undergo menstrual disorder (characterized as amenorrhea, oligomenorrhea or polymenorrhea) and infertility [1–4]. This situation can hardly be improved by hemodialysis (HD) but can be ameliorated after renal transplantation (RT) [3, 5–9]. It is demonstrated that the impaired positive estradiol (E_2_) feedback and the absence of luteinizing hormone (LH) surge cause anovulation and menstrual irregularity during HD [8, 10]. After RT, women can resume normal gonadal hormone secretion patterns [5] and regular menses [3, 9, 11]. However, there is residual dysfunction of reproductive system, which is accused of the immunosuppressive agents and corticosteroids used after RT [5, 12]. Studies suggest that the dysfunction of hypothalamic-pituitary gonadal axis is partly caused by highly elevated prolactin (PRL) concentration since with the normalization of this hormone, LH surge, menses and ovulation can regain, to some extent [10, 13].

But the recovery of menstruation doesn’t mean the recovery of hypothalamic-pituitary function. The presence of anovulatory cycles and luteal phase deficiency in HD women still menstruating [5, 14] suggest that we need to take further attention on hormone status. Studies have shown that HD women have a diverse secretion pattern of gonadal hormones [8, 10, 15]. Handelsman D.J. at al. [8] reviewed the variable hormones of HD women, but they didn’t concentrated on the cause and hormone status after RT, and failed to give a quantitative analysis.

Hormone derangement in HD women not only is a reflection of reproductive disorder, but also has relationship with life quality and survival [16]. Konstantinos K. et al. [17] considered that bone mineral density was negatively correlated with follicle-stimulating hormone (FSH) values while positively associated with E_2_ values in a cohort study with 21 HD women and 21 HD men. Tanrisev, M. et al. [18] found that E_2_ had a U-shaped correlation with cardiovascular and overall mortality in postmenopausal HD women. As for sexual function, E_2_ is also associated with arousal, orgasm and pain in HD women [19]. While for PRL, Carrero JJ et al. [20] concluded that its value was positively associated with endothelial dysfunction, cardiovascular and all-cause mortality in both nondialyzed and HD patients.

Furthermore, to our knowledge, there is no meta-analysis concluding feminine hormones in HD women. We focus on HD women, with healthy and post-RT women as controls, to perform a meta-analysis to show a whole hormone profile in HD women as further prognosis information and to explore if menses status, PRL or other factors should play a role in the changes of those hormones.

## Methods

### Search strategy and study selection

Relevant search strategies were in accordance with the PICOS framework and showed as follows: (I) study population (Chronic renal failure, Uremia*, Chronic Kidney Failure, Blood Urea Nitrogen); (II)exposure (Hemofiltration or Renal Dialysis*); (III) outcomes (Gonadal Hormones, Gonadal Steroid Hormones, Sexual Hormones, and prolactin, STable 1). The comparison (renal transplantation and healthy control) was omitted to get more simplified results. Keywords were adjusted and the wildcard characters such as * was used in search terms of sexual*, hormon*, h?emofiltrat* and renal* for a second search to yield more comprehensive results. Key words were also adjusted when searched in different databases. The references of related articles were also screened to conduct backward and forward snowballing searches to identify additional relevant articles. Authors of potentially eligible articles were contacted for further information.

After merging citations and eliminating duplications manually, two reviewers screened titles and abstracts independently to obtain eligible articles. Studies potentially containing relevant data were retained initially. A third author joined to make a consensus when disagreement occurred. The study was registered in PROSPERO to further enhance the transparency and to better explain issues arising during research. The register number was CRD42023394503.

### Inclusion and exclusion criteria

Those articles were included: (I) The study group was women with chronic renal failure undergoing regular HD and the control group was age-matched healthy women or women after kidney transplantation at least 6 months with stable renal function; (II) Studies presented data about any of the following hormones: FSH, LH, E_2_, progesterone(P) and PRL; (III) Initial data was available or data were shown as mean ± standard deviation or standard error of the mean (mean ± SD/SEM) or median and range/interquartile range; (IV) Articles reported in English without limitation of study design. Studies were excluded with the following criteria: (I) Insufficient data; (II) Mixed data of female and male; (III) Case report or review. Studies were divided into subgroup 1 (blood samples taken during follicular phase) and subgroup 2 (blood samples taken during the other time or not mentioned).

### Data extraction and quality evaluation

Two reviewers independently extracted information from identified articles using a standardized data extraction form including first author, publication year, country, sample size, age, hormones levels, units and factors which might influence hormone concentrations, such as menses, the serum creatinine levels, medicine, time taking blood samples, HD duration, etc. If subgroups were present within included studies, we only extracted data from the relevant subgroups. Different subgroups were merged if necessary. If only original data were presented in articles, mean value and standard deviation would be calculated after normality test. In one study [21] which had hormones tested 1 to 6 times at the start of the mid-menstrual circle, we took the mean value as a representative of the individual. If the control group had hormones tested during follicular phase, mid-cycle and luteal phase, the follicular phase values were taken as control. If studies were classified as intervening cohort study, hormones values of HD and healthy control prior to the intervention were collected. Data shown as median and range/interquartile range were transformed to mean and standard deviation by online tool (https://smcgrath.shinyapps.io/estmeansd/).

We used the risk of bias in non-randomised studies of exposures (ROBINS-E) tool [22] and online tool GRADEpro GDT to assess the quality of included studies. Two authors assessed the articles and any discrepancy was discussed and resolved with the third author. A kappa statistic was used to demonstrate agreement between the authors.

### Statistical analysis

Data collected (PRL, FSH, LH, E_2_, P) were divided into three parts: first, hormones of HD women versus those of age-matched healthy controls (HD vs. Ct group); second, hormones of HD women versus those of age-matched healthy control or women after RT (HD vs. RT group); third, comparison of hormones between women undergoing HD with or without menstrual disorder (HDre vs. HDir). We used Review Manager 5.4, STATA (version 14.0) and trial sequential analysis (TSA) software (TSA 0.9.5.10 Beta) for data analysis. Results were shown as standardized mean difference (SMD) and 95% confidence interval (95%CI) in this meta-analysis because of not mentioning testing methods and inconsistent units in different studies. The heterogeneity of included studies was evaluated by *I*^2^ test and tau^2^. In light of the substantial heterogeneity observed across various comparisons, we selected the random effect model (I-V (instrumental variables) heterogeneity model in STATA14) for this meta-analysis, as it proves more fitting for addressing the inter-study heterogeneity. Meta-regression analysis was employed to explore the correlations between gonadal hormones and other factors such as mean patient age, PRL and serum creatinine per study if there were available data and at least 10 studies included in analysis. The publication bias was evaluated by Egger’s test. Meta-trim analysis, which added some invented studies to fill the funnel plot to make it symmetrical and then to do meta-analysis for all studies to test if the overall effect was changed, was used to test the conclusion stability if bias existed. The conclusion robustness and reliability were tested by sensitivity analysis, in which the synthesized effects after excluding the study on the left were represented by dots and their confidence intervals were represented by horizontal lines. When the line was outside the confidence interval of the overall results or caused a significant change in the synthesized effect size, it indicated that the study had a major impact on the results.

According to the characteristics of the data, we added TSA analysis to detect the possibly existed small study effects and to enhance the conclusiveness of meta-analysis. In the TSA analysis, all hormone values were regarded as negative factors, so, the favoring group in TSA graph signified a lower hormone value. There were 5 important lines in the TSA graph including Z curve, the conventional boundary, monitoring boundary, futility boundary and the trial sequential monitoring boundaries (required information size) [23]. The method of TSA analysis was followed by User manual for Trial Sequential Analysis (www.ctu.dk/tsa).

## Results

### Search results

**Fig. 1:**
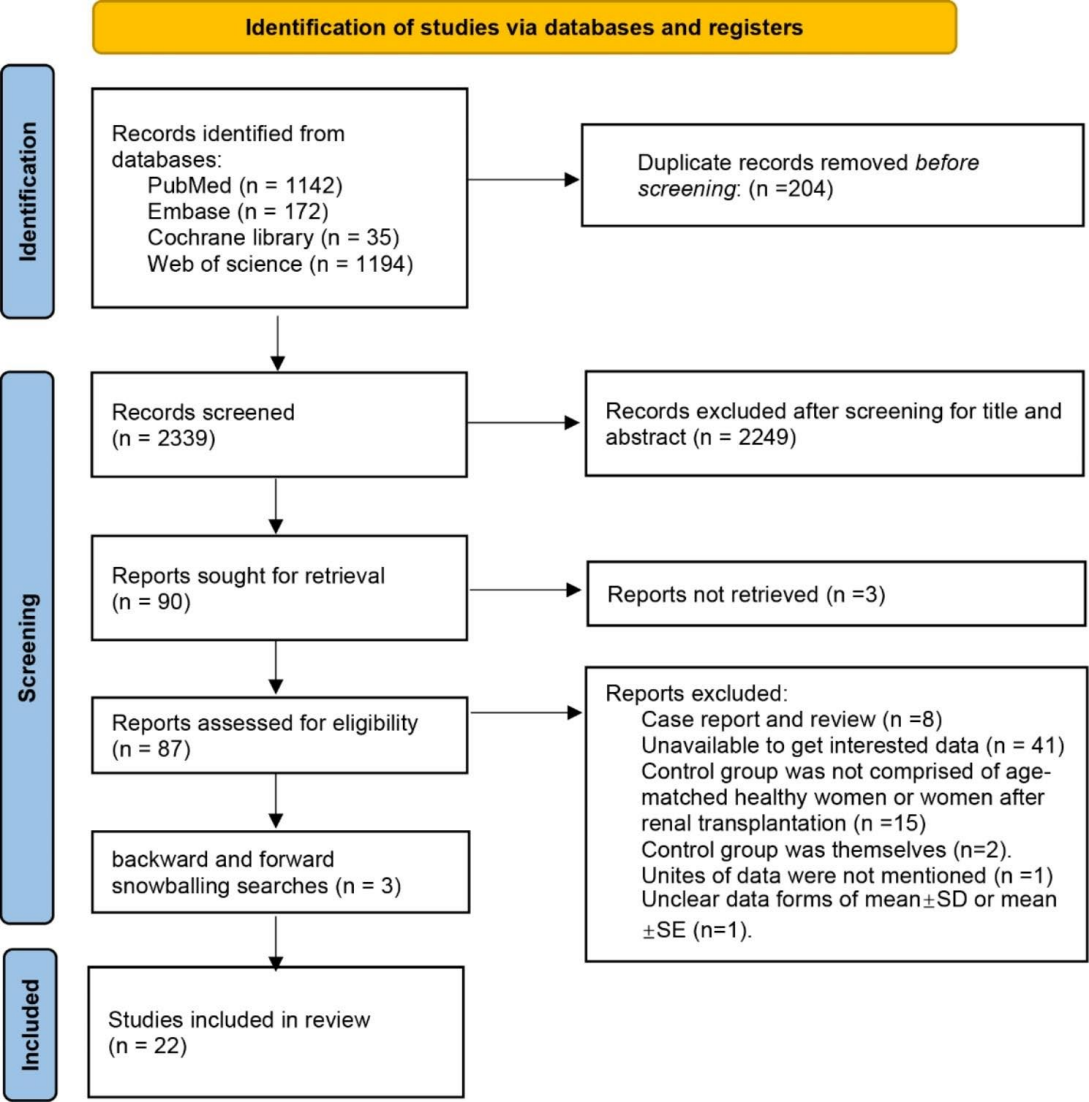
Flow chart about screening and including literature

A total of 2543 articles were found and 2339 remained after duplicates removed. Then, 87 full-text articles were kept and assessed for eligibility after excluding irrelevant studies by screening titles and abstracts. According to inclusion and exclusion criteria, 3 case reports and 5 reviews were excluded. There were 41 articles excluded for unavailable interested data, of which 20 studies reported data of male or a mixture of male and female. The remaining 21 articles didn’t contain data of relevant hormones or data were insufficient. Fifteen articles were removed because control group was not comprised of age-matched healthy women or women after RT. Two cohort studies were also excluded as they did not include control groups at baseline and instead, they opted to compare participants with themselves six months after RT [12, 24]. We also excluded a study without the units of data as well as a study that did not specify whether the data were presented as mean ± SD or mean ± SEM [25]. We adjusted keywords and conducted backward and forward snowballing searches and found 3 extra articles. Finally, a total of 22 articles [1, 10, 19, 21, 26–43] were included in this meta-analysis. A PRISMA 2020 flowchart was provided to illustrate the screening and including process (Fig. 1). To show the inter-rater reliability of the data screening and selection process, we added kappa statistic and the kappa value was 0.799, showing a high level of consistency between the authors.

### Study characteristic

Of the included 22 articles from 13 countries (STable 2), 17 articles [10, 19, 26–35, 38, 40, 42, 43] compared hormone profiles of HD women to age-matched healthy controls (Ct) and 7 articles [1, 26, 27, 31, 38, 39, 41] compared hormone features of HD women to women after RT. What’s more, 7 articles [1, 21, 29, 30, 36, 37, 43] provided hormones data of age-matched premenopausal HD women with or without regular menses (HDre vs. HDir). As a means to reduce heterogeneity among the studies, we created a subgroup consisting of studies where blood samples were obtained during the follicular phase of the menstrual cycle.

### Quality assessment

All studies were assessed by ROBINS-E tool (STable 3 to STable 5, results represented low to moderate risk of bias) and the online tool GRADEpro GDT (Stable 6 to Stable 8, except the E_2_ and P in HD versus RT group, all got moderate certainties). All patients were identified after a regular HD or after RT at least 6 months. Three articles [19, 33, 34] included consecutive subjects. Other than 4 [19, 31–33] studies, all described the method for hormone measurement. Eight studies [1, 26, 27, 29, 30, 34, 35, 43] took the blood samples of menstruating women during the follicular phase. Other 14 studies took blood samples of all subjects at the same time of a random day [19, 32, 33, 41] or before HD [28, 31] or others [21, 36, 42] or not mentioned [10, 37–40]. There were 12 articles [1, 10, 19, 21, 28–30, 33, 36, 37, 42, 43] excluding the medicine interfering for hormones assessment, which was one of the major factors for confounding assessment. All participants had needed hormones measured but one study [10] which had 2 normal controls failed to complete hormones measurement and the remaining participants were taken as controls.

### Hormone levels of HD women compared with that of normal control (HD vs. ct)

Subgroup analysis was not performed in PRL values comparison as it was considered having little to do with the menstrual circle.

**Fig. 2:**
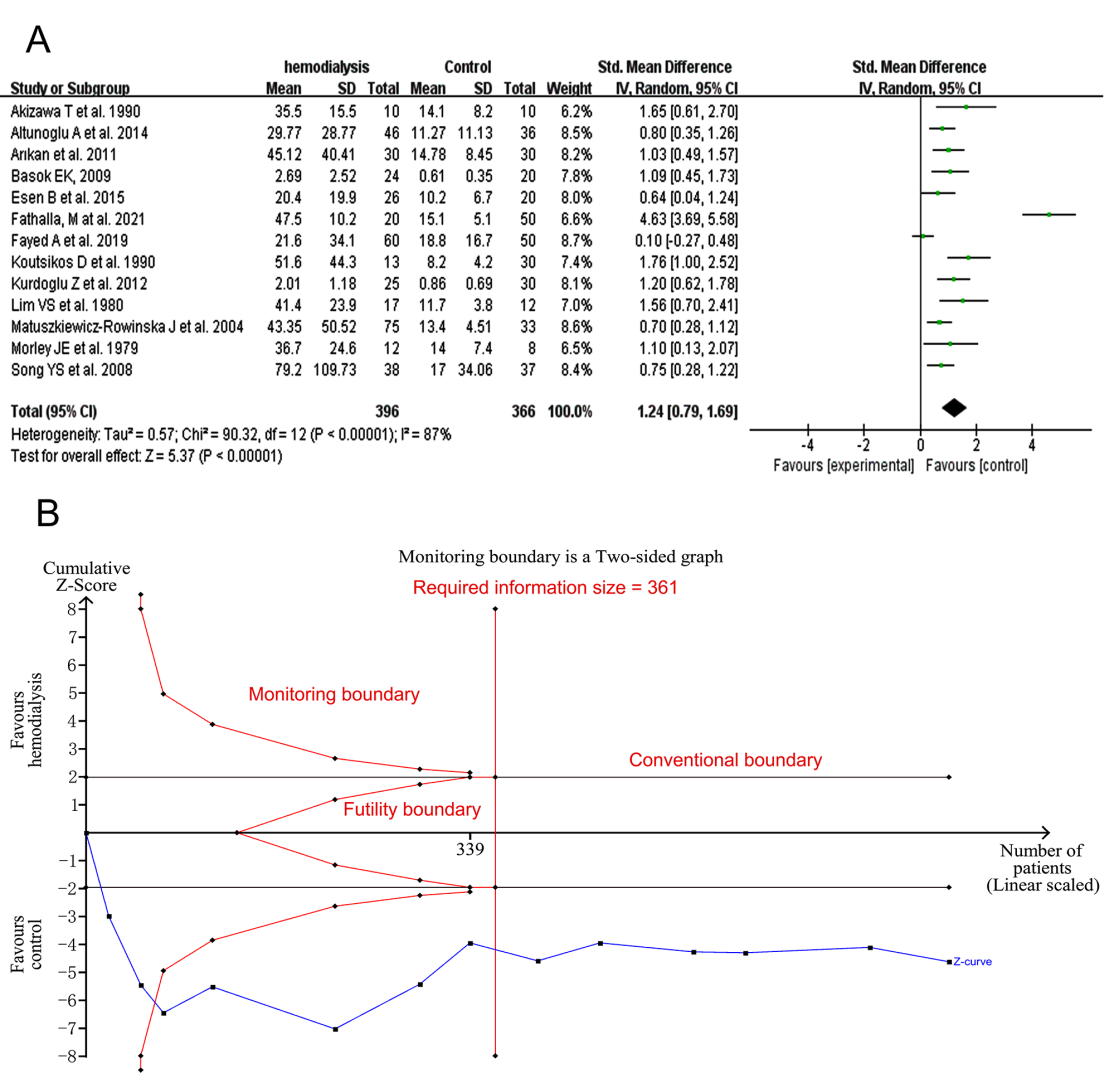
Forest plot and TSA analysis of prolactin in HD vs. Ct group. **A**, forest plot of prolactin; **B**, TSA analysis of prolactin. HD, hemodialysis; Ct, control

Serum PRL level was significantly higher in HD women than that of control group (*I*^*2*^ = 87%, tau^2^ = 0.57, SMD 1.24, 95% CI: 0.79–1.69, Fig. 2A). When the one study with large heterogeneity was excluded, the *I*^*2*^ reduced to 62% and the result remained unchanged. Meta-trim analysis was used to test conclusion stability for still existed publication bias (Egger’s test, *p* = 0.0020) when the study of Fathalla, M at al. showing large heterogeneity was excluded, and the result not changed when 5 invented studies were added to make the funnel graph symmetrical (*p* = 0.000 in random effects mode). The sensitivity analysis was showed in SFigure 1. We wanted to do a meta-regression analysis to test if the mean age of per study could contribute some heterogeneity, but we couldn’t get the actual mean age of all included studies’, therefore, we did a meta-regression analysis between PRL values and the mean age of HD women without the study of Lim VS et al. for the unavailable mean age. And the result of meta-regression showed no statistical significance (*p* = 0.899, SFigure 2). In the TSA analysis of PRL shown in Fig. 2B, the Z curve stayed out of futility borders, monitoring boundaries and reached the required information size both before (Fig. 2B) and after (SFigure 3) excluding the study aforementioned showing large heterogeneity.

**Fig. 3:**
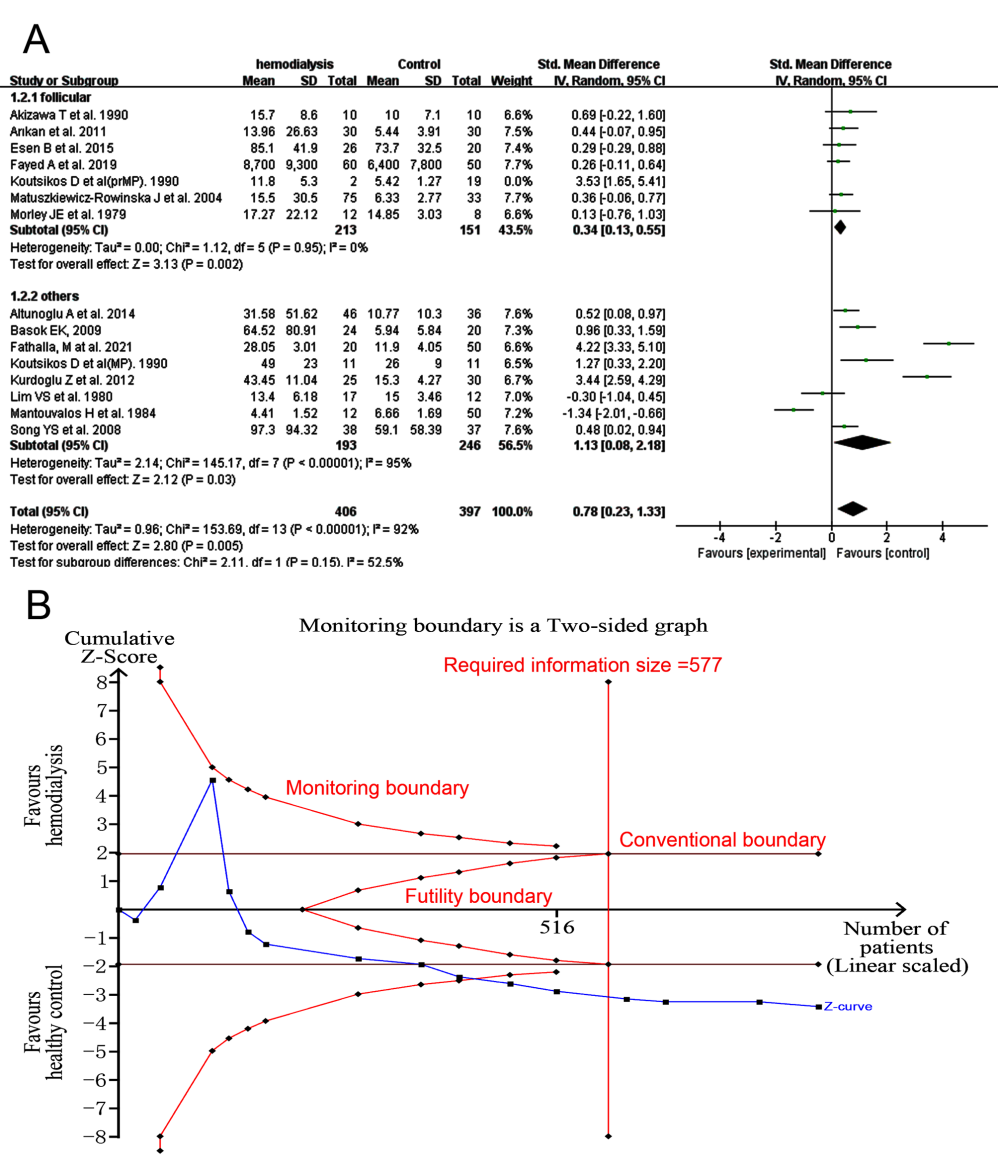
Forest plot and TSA analysis of follicle-stimulating hormone (FSH) in HD vs. Ct group. **A**, forest plots of FSH after excluding the study of Koutsikos D et al. (Koutsikos D et al(prMP)); **B**, TSA analysis of FSH.

Serum FSH was higher in hemodialysis women in subgroup 1, subgroup 2 and overall (*I*^*2*^ = 0%, tau^2^ = 0, SMD 0.34, 95% CI:0.13–0.55, *p* = 0.002 in subgroup 1; *I*^*2*^ = 95%, tau^2^ = 2.14, SMD 1.13, 95% CI: 0.08–2.18, *p* = 0.03 in subgroup 2; *I*^*2*^ = 92%, tau^2^ = 0.96, SMD 0.78, 95% CI: 0.23–1.33, *p* = 0.005 as a whole, Fig. 3A) after excluding the premenopausal women in the study of Koutsikos D et al. (Koutsikos D et al(prMP)) which showed a significant heterogeneity in subgroup1. Results of all included studies before exclusion was shown in SFigure 4. No publication bias existed (Egger’s test, *p* = 0.0954). The sensitivity analysis of all included studies was shown in SFigure 5. Meta-regression showed that there was no statistical significance between mean age of HD women and hormones values (*p* = 0.974, SFigure 6). In the TSA analysis of FSH values in follicular phase, the Z curve stayed within the conventional boundary, out of the futility boundary, and the required information size boundary was ignored due to limited number of studies (SFigure 7). The Z curve of all included studies finally stayed out of the monitoring boundary and the futility boundary (Fig. 3B).

**Fig. 4:**
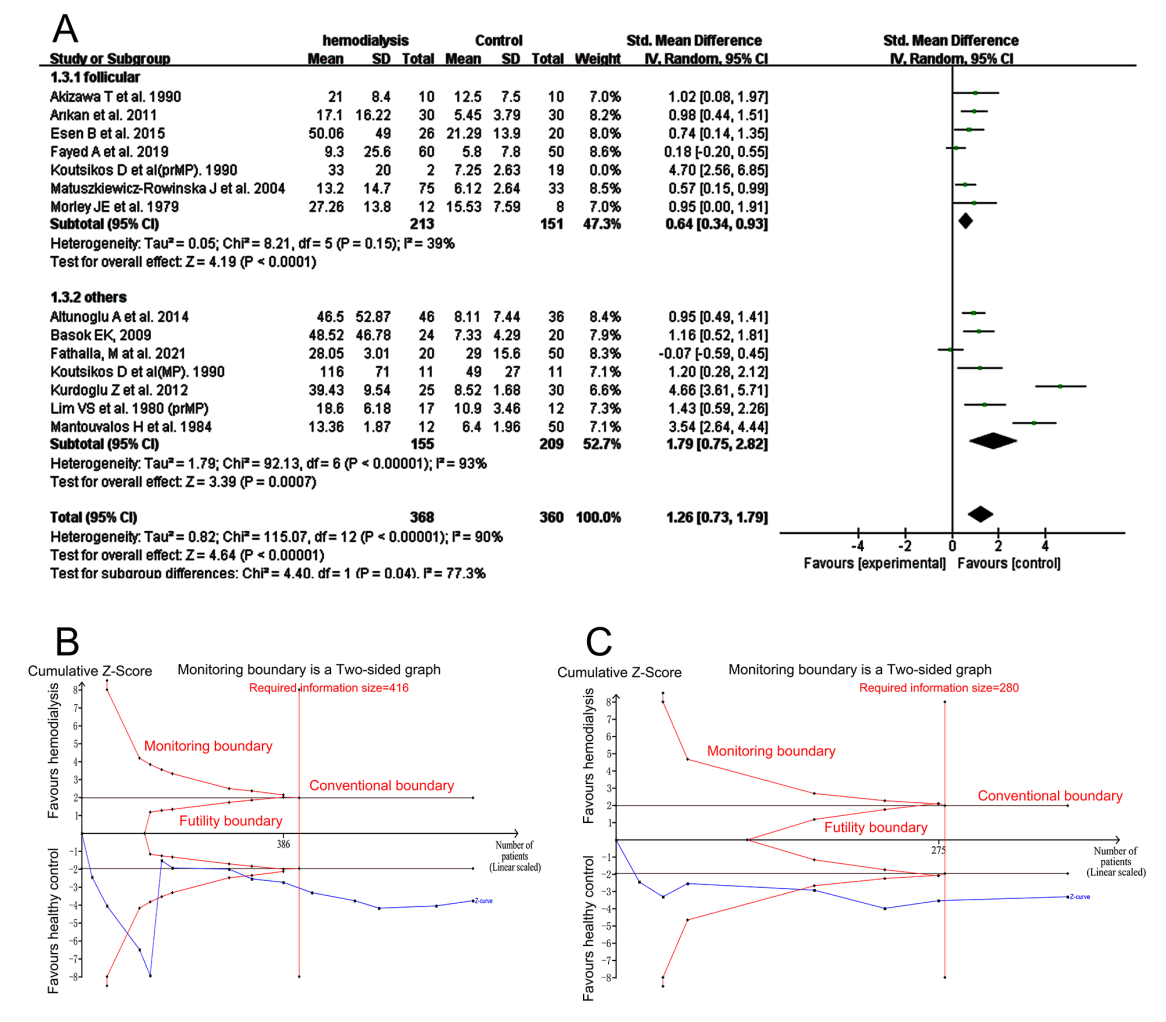
Forest plot and TSA analysis of luteinizing hormone (LH) in HD vs. Ct group. **A**, forest plot of LH after excluding the study of Koutsikos D et al. (Koutsikos D et al(prMP)) and Cengiz K et al.; **B**, TSA analysis of LH of all included studies; **C**, TSA analysis of LH tested during follicular phase

Serum LH (Fig. 4A) was higher in HD women (*I*^*2*^ = 39%, tau^2^ = 0.05, SMD 0.64, 95% CI: 0.34–0.93, *p* < 0.0001 in subgroup 1; *I*^*2*^ = 93%, tau^2^ = 1.79, SMD 1.79, 95% CI: 0.75–2.82, *p* = 0.0007 in subgroup 2; *I*^*2*^ = 90%, tau^2^ = 0.82, SMD 1.26, 95% CI: 0.73–1.79, *p* < 0.00001 as a whole) after excluding the study of Koutsikos D et al. (Koutsikos D et al.(prMP)) showing large heterogeneity. Results before any exclusion was presented in SFigure 8. There was publication bias overall (Egger’s test, *p* = 0.0028) and after the study aforementioned was excluded (Egger’s test, *p* = 0.0028). So, we did trim and fill analysis and the statistical significance was not changed overall (*p* = 0.036 in random effects mode) and in subgroup1(*p* = 0.028 in random effects mode). Sensitivity analysis was showed in SFigure 9. Meta-regression analysis presented no statistical significance between mean age of HD women in per study and LH values (*p* = 0.471 for all included studies, SFigure 10). Analysis of TSA showed that the Z curve of LH values in subgroup 1 and overall stayed out of futility borders, monitoring boundaries and reached the required information size (Fig. 4B and C).

Serum E_2_ values were similar in HD women in follicular phase (subgroup 1) before (*I*^*2*^ = 81%) and after (*I*^*2*^ = 21%, tau^2^ = 0.03, SMD − 0.10, 95% CI: -0.40-0.20, *p* = 0.52, SFigure 11) excluding the premenopausal women of the study of Koutsikos D et al. (Koutsikos D et al.(prMP)) in subgroup1. Plasma E_2_ values were similar between HD women and healthy control before excluding the study aforementioned (*I*^*2*^ = 90%, tau^2^ = 0.88, SMD − 0.40, 95% CI: -0.91 to 0.12, *p* = 0.13, SFigure 12). Egger’s test showed no publication bias in subgroup 1 (*p* = 0.2689) and in overall studies (*p* = 0.9942). Sensitivity analysis was showed in SFigure 13. There was no statistical significance (*p* = 0.970) between the mean age of HD women in per study and E2 values in meta-regression analysis (SFigure 14). In the TSA analysis testing for small study effects, the required information size boundary was ignored in the graph due to not enough of studies (SFigure 15).

**Fig. 5:**
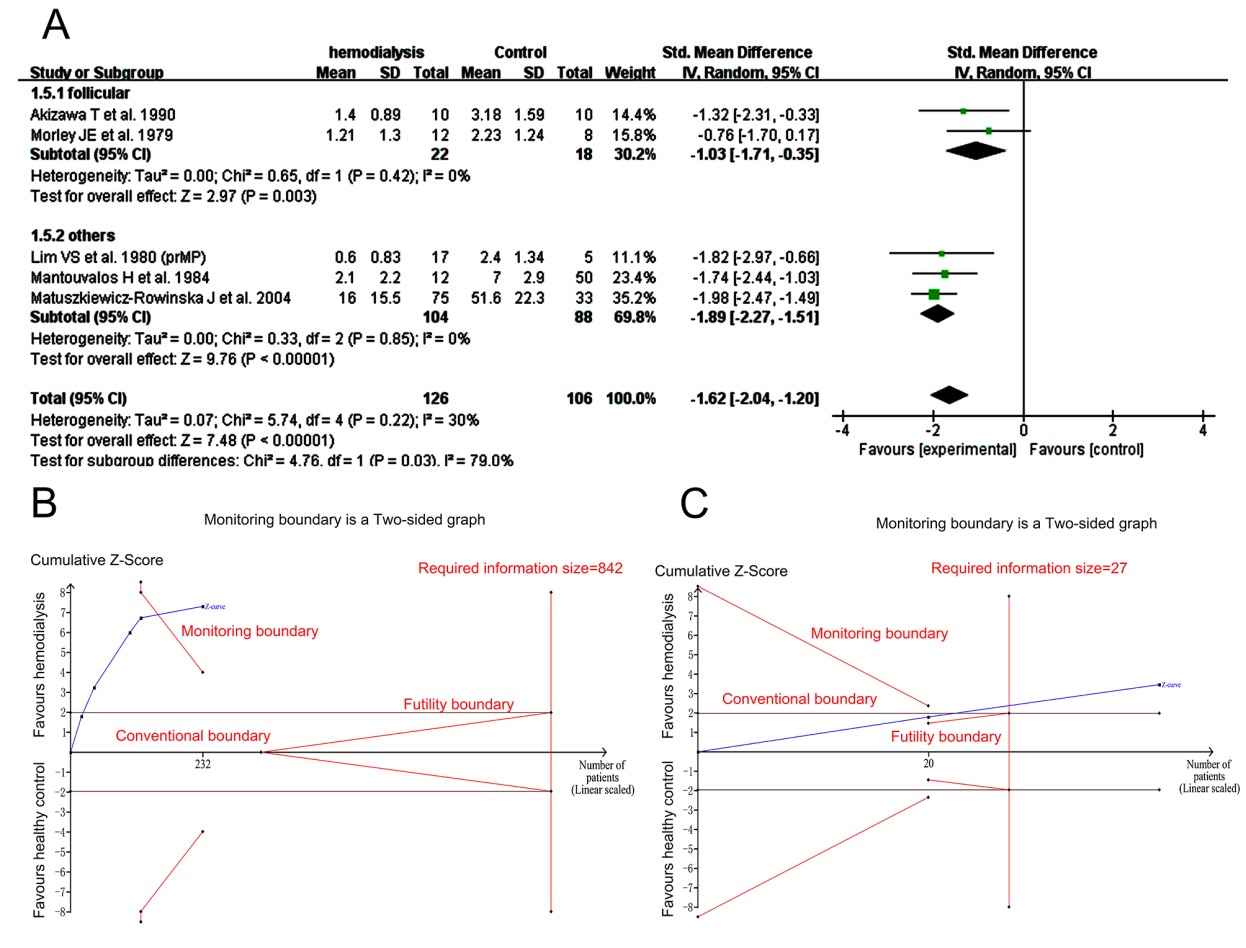
Forest plot and TSA analysis of progesterone (P) in HD vs. Ct group. **A**, forest plot of P values; **B**, TSA analysis of P values of all included studies. **C**, TSA analysis of P values tested during follicular phase

Serum P level was lower than that of the control tested in the follicular phase (*I*^*2*^ = 0%, tau^2^ = 0.00, SMD − 1.03, 95% CI: -1.71 to -0.35, *p* = 0.003) and in the subgroup 2 (*I*^*2*^ = 0%, tau^2^ = 0.00, SMD − 1.89, 95% CI: -2.27 to -1.51, *p <* 0.00001) and overall (*I*^*2*^ = 30%, tau^2^ = 0.07, SMD − 1.62, 95% CI: -2.04 to -1.20, *p <* 0.00001, Fig. 5A). There was no publication bias (Egger’s test, *p* = 0.2197). Sensitivity analysis was showed in SFigure 16. The Z curve both in overall (Fig. 5B) and in subgroup 1 (Fig. 5C) stayed out of the conventional boundary and the monitoring boundary or reached the required information size in TSA analysis.

### Hormone levels of HD women compared with that after RT (HD vs. RT)

**Fig. 6:**
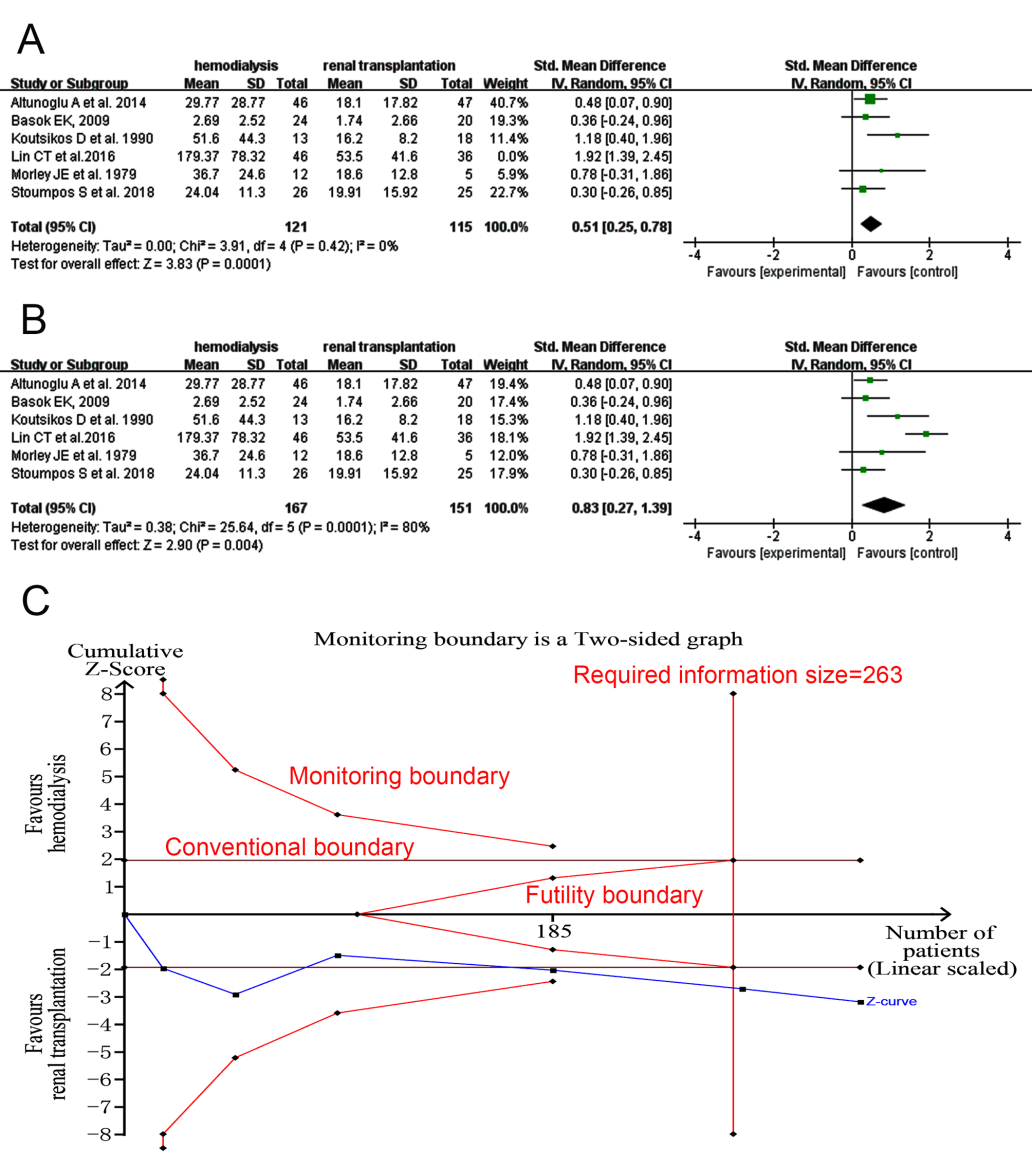
Forest plot and TSA analysis of prolactin (PRL) in HD vs. RT group. **A**, forest plot of PRL after excluding the study of Lin CT et al. showing large heterogeneity; **B**, forest plot of PRL of all studies; **C**, TSA analysis of PRL of all included studies

Serum PRL level was higher in HD women (*I*^*2*^ = 0%, tau^2^ = 0.00, SMD 0.51, 95% CI: 0.25 to 0.78, p = 0.0001; Fig. 6A) after and before (*I*^*2*^ = 80%, tau^2^ = 0.38, SMD 0.83, 95% CI: 0.27–1.39, *p* = 0.004; Fig. 6B) excluding the study of Lin CT et al. showing large heterogeneity. No publication bias was presented (Egger’s test, *p* = 0.3327 after and *p* = 0.7883 before excluding the study aforementioned). Sensitivity analysis was showed in SFigure 17. In TSA analysis, the Z curve stayed out of the conventional boundary, the futility boundary and reached the required information size (Fig. 6C).

Serum FSH level in HD women was higher than that of RT women sampled in the follicular phase (*I*^*2*^ = 0%, tau^2^ = 0.00, SMD 0.96, 95% CI: 0.55–1.37, *p <* 0.00001 in subgroup 1) and overall (*I*^*2*^ = 59%, tau^2^ = 0.15, SMD 0.49, 95% CI: 0.10–0.89, *p* = 0.02 as a whole, SFigure 18), but was similar to that of RT women in subgroup 2 (*I*^*2*^ = 61%, tau^2^ = 0.14, SMD 0.30, 95% CI: -0.18-0.78, *p* = 0.22). Sensitivity analysis showed in SFigure 19 and Egger’s test exhibited no publication bias overall (*p* = 0.7385). The monitoring boundary was ignored due to limited number of studies included in TSA analysis (SFigure 20).

Serum LH concentration was also higher in HD women (*I*^*2*^ = 0%, tau^2^ = 0.00, SMD 1.08, 95% CI: 0.59–1.57, *p <* 0.0001 in subgroup 1; *I*^*2*^ = 59%, tau^2^ = 0.13, SMD 0.42, 95% CI: -0.05-0.88, *p* = 0.08 in subgroup 2; *I*^*2*^ = 59%, tau^2^ = 0.17, SMD 0.64, 95% CI: 0.22–1.06, *p =* 0.003 as a whole, SFigure 21). No publication bias existed (Egger’s test, *p* = 0.2596) and sensitivity analysis was showed in SFigure 22. In TSA analysis, the Z curve was unable to reach the monitoring boundary, the futility boundary and the required information size (SFigure 23).

No significance was found in serum E_2_ level between HD and RT group in subgroup1 and overall (*I*^*2*^ = 93%, tau^2^ = 14.85, SMD 1.54, 95% CI: -3.99-7.07, *p* = 0.58 in subgroup 1; *I*^*2*^ = 81%, tau^2^ = 0.37, SMD − 0.66, 95% CI: -1.26 to -0.05, *p* = 0.03 in subgroup 2; *I*^*2*^ = 83%, tau^2^ = 0.60, SMD − 0.47, 95% CI: -1.14 to 0.20, *p* = 0.17 as a whole, SFigure 24) with sensitivity analysis shown in SFigure 25. Egger’s test showed no publication bias (*p* = 0.3723). The Z curve was within the conventional boundary and the monitoring boundary was ignored due to insufficient studies included (SFigure 26).

Serum P concentration was similar between HD women and women after RT (*I*^*2*^ = 87%, tau^2^ = 0.66, SMD − 0.31, 95% CI: -1.32-0.70, *p =* 0.54, SFigure 27) with no publication bias (Egger’s test, *p* = 0.8433). Sensitivity analysis was omitted. In addition, the Z curve of TSA analysis stayed within the conventional boundary and the monitoring boundary and the required information size were ignored for few studies included (SFigure 28).

### Hormone levels between HD women with or without regular menses (HDre vs. HDir)

Serum PRL levels were lower in women with regular menses (*I*^*2*^ = 91%, tau^2^ = 1.36, SMD − 1.37, 95% CI: -2.45 to -0.28, *p* = 0.01, SFigure 29). When the study of Lin CT et al. was excluded, there was good homogeneity without changing the overall effect (*I*^*2*^ = 41%, tau^2^ = 0.07, SMD − 0.60, 95% CI: -1.02 to -0.18, *p* = 0.005, SFigure 30). Egger’s test showed no publication bias existed (*p* = 0.0725). Sensitivity analysis was showed in SFigure 31. The Z curve stayed within the conventional boundary, out of the futility boundary and didn’t reach the required information size in TSA analysis (SFigure 32).

FSH concentration was similar between regular and irregular menses women both in subgroup 1 and subgroup 2 (*I*^*2*^ = 85%, tau^2^ = 0.59, SMD − 0.78, 95% CI: -1.60-0.05, *p* = 0.06 in subgroup 1; *I*^*2*^ = 0%, tau^2^ = 0.00, SMD − 0.39, 95% CI: -0.78 to -0.00, *p* = 0.05 in subgroup 2, SFigure 33). Sensitivity analysis showed that there was large heterogeneity in the study of Lin CT et al. (SFigure 34). After excluding the study of Lin CT et al. [1], we got a good homogeneity in subgroup 1 (*I*^*2*^ = 0%, tau^2^ = 0.00, SMD − 0.29, 95% CI: -0.61-0.04, *p* = 0.08, SFigure 35). But serum FSH was higher in irregular hemodialysis women in overall both before (*I*^*2*^ = 74%, tau^2^ = 0.30, SMD − 0.55, 95% CI: -1.04 to -0.06, *p* = 0.03) and after (*I*^*2*^ = 0%, tau^2^ = 0.00, SMD − 0.33, 95% CI: -0.58 to -0.08, *p* = 0.009) excluding the study aforementioned. Egger’s test didn’t present publication bias before (*p* = 0.6203) and after (*p* = 0.5449) excluding the study of Lin CT et al. In TSA analysis, the Z curve was within the conventional boundary, out of the futility boundary and didn’t reach the required information size (SFigure 36).

Serum LH level was lower in HD women with regular menses in subgroup1 and overall (*I*^*2*^ = 53%, tau^2^ = 0.11, SMD − 0.62, 95% CI: -1.07 to -0.17, *p* = 0.007 in subgroup1; *I*^*2*^ = 34%, tau^2^ = 0.05, SMD − 0.48, 95% CI: -0.78 to -0.17 in overall, SFigure 37). But serum FSH was comparable between HD women with or without regular menses in subgroup2 (*I*^*2*^ = 0%, tau^2^ = 0.00, SMD − 0.25, 95% CI: -0.64 to -0.14, *p* = 0.20). Sensitivity analysis was showed in SFigure 38. There was no publication bias existed by Egger’s test (*p* = 0.2856). The Z curve in TSA analysis failed to reach the monitoring boundary, the futility boundary and required information size (SFigure 39).

Subgroup analysis was not performed in this comparison. Result exhibited that serum E_2_ was higher in regular menses women both before (*I*^*2*^ = 96%, tau^2^ = 3.82, SMD 2.13, 95% CI: 0.17–4.09, *p* = 0.03, SFigure 40) and after (*I*^*2*^ = 0%, tau^2^ = 0.00, SMD 0.87, 95% CI: 0.48–1.26, *p <* 0.0001, SFigure 41) excluding the study of Weisinger JR et al. There existed no publication bias by Egger’s test (*p* = 0.2977). The sensitivity analysis presented in SFigure 42. The Z curve of TSA analysis didn’t reach the monitoring boundary, the futility boundary and required information size (SFigure 43).

Serum P values in this part were all tested during luteal phase and were higher in women with regular menses (*I*^*2*^ = 50%, tau^2^ = 0.11, SMD 1.23, 95% CI: 0.58–1.88, *p* = 0.0002, SFigure 44). Sensitivity analysis and Egger’s test were omitted because of only 2 studies included in this part. In TSA analysis, the monitoring boundary and required information size were omitted due to few studies included (SFigure 45).

## Discussion

### Brief summary

In HD vs. Ct group, serum PRL, FSH and LH values were higher in HD women while the plasma P value was lower in HD women. Serum E2 concentration tested during follicular phase were comparable to normal control and varied from low to normal overall, but this conclusion needed further confirmation. In HD vs. RT group, we found higher serum PRL values in HD women. What’s more, higher FSH and LH levels and lower serum P level in HD women were presented and serum E2 level was comparable to that of women after RT, but they needed further confirmation due to not reaching the monitoring boundary or the futility boundary or required information size in TSA analysis. In HDre vs. HDir group, there were lower PRL, LH values, similar follicular FSH levels, higher E2 and P levels in regular menses HD women and they all needed conformation because of not reaching the monitoring boundary, the futility boundary or required information size in TSA analysis.

### Measures to adjust small-study effects

A considerable number of studies with small sample sizes were included in this meta-analysis, In order to adjust small-study effects, we did trim-and-fill analysis to confirm the conclusion stability if publication bias existed and the results presented good stability of conclusion in this study. What’s more, we did TSA analysis to further evaluate the precision and uncertainty of the results in our study [23]. In TSA analysis, if the Z-curve stayed within the conventional boundary and out of the futility boundary, or the Z-curve stayed out of the conventional boundary, within the monitoring boundary and not reaching the required information size, it meant the conclusion of meta-analysis needed further confirmation. While we could draw the conclusion that there was no statistical significance if the Z-curve reached the futility boundary. And if the Z-curve stayed out of the monitoring boundary or it reached the required information size, we were able to conclude that there was statistical significance [23]. Those two measures enhanced the credibility and reliability of the conclusions in our study.

### Measures to bolster the conclusion validity

To further enhance the credibility of the conclusion and internal validity in our study, we implemented the following measures. Firstly, strict criteria for study inclusion and exclusion were set to maintain the internal validity and a thoroughly search was performed to avoided selective reporting. Secondly, a standardized tool (ROBINS-E tool) was applied to assess the quality of included studies and results presented a moderate to high quality of all studies included (STable 3 to STable 5). Thirdly, two separate authors extracted data to maintain data consistency and there was high consistency (kappa = 0.799). In addition, sensitivity analysis was down to assess the stability of the results and mitigate potential bias due to the interference to the results caused by some particular studies. What’s more, we conducted subgroup and meta-regression analyses to explore potential sources of heterogeneity. And there was a relatively small heterogeneity in subgroup 1(sampling during follicular phase) in this study. Researches found that about l% to 10% HD women [3, 11, 44] had regular menses and part of them still existed cyclic hormone secretion [5] which proved the necessity of subgroup analysis. Fathalla, M at al. [42] found that the age of patients was positively correlated with plasma PRL, LH, and FSH in HD women. But we couldn’t obtain statistical significance in meta-regression analysis.

### Main research findings

PRL values were consistently elevated in HD women compared with normal control and women after RT. It was possibly caused by diversely increased PRL secretion rate due to CKD-mediated inhibition of dopaminergic activity [45] and differently decreased PRL elimination by kidney [46]. Moreover, a higher FSH concentration was found in HD women compared with normal control in subgroup 1 and the unclear sampling time largely contributed to the diversified FSH levels overall. Furthermore, serum P levels tested during follicular phase and luteal phase were lower in HD women in this study, demonstrating no ovulation in HD women.

We found consistent elevation of LH and PRL levels in HD women which could be caused by decreased elimination [47]. We supposed that the elevated LH level was also caused by an increased secretion. First, concomitant rising episodes and a positive linear correlation (r = 0.74, *p* < 0.001) of pulses frequency of the PRL and LH were found during menstrual cycles in healthy women [48, 49]. And in anatomy [50], it was demonstrated that kisspeptin neurons in hypothalamus regulated PRL and LH secretion simultaneously. Second, in HD women, the PRL increased primarily by increased secretion [45], and PRL and LH had consistent elevation as aforementioned. Those mentioned above gave us clues to deduce that LH also had an increased secretion in HD women. Schaefer F et al. [51] demonstrated that the immunoreactive LH enhanced production, with dramatically decreased bioactive LH level, leading to significantly elevated mean LH concentration in HD children comprised of 18 boys and 18 girls supporting our hypothesis that LH, like PRL, had an increased secretion in HD women.

The hormone status except PRL values in HD vs. RT group and hormones in HDre vs. HDir group needed further confirmation according to the TSA results. Study showed that gonadotropin could be ameliorated after RT [26], which was in accordance with the meta-analysis results in this study. Filocamo MT et al. [24] found that concentrations of FSH, LH and E_2_ were not changed during HD and 1-year after RT in menstruating HD women, but FSH, LH were all decreased and E_2_ were increased1-year after RT in non-menstruating HD women indicating that RT ameliorated disturbed hormones status of HD women, most probably of those without regular menses.

### The interference of adrenal and thyroidal hormones

Abnormal hypothalamic pituitary adrenal (HPA) and thyroid axis (HPTA) also affected gonadal hormones in HD women. Studies suggested that adrenocorticotrophic (ACTH) hormone values variated from normal to high and cortisol levels were elevated in HD patients [8]. And HPA hormones were thought to inhabit the secretion of gonadotropin-releasing hormone (GnRH), LH, E_2_ and P in hypothalamus pituitary ovary (HPO) axis [52], which could be one of the reasons of the diversified levels of gonadal hormones and anovulation in HD women. In addition, thyroid stimulating hormone (TSH) and free thyroxine (fT_4_) values ranged from low to high and free triiodothyronine (fT_3_) levels were decreased in HD women [8]. It was considered that altered thyroid hormones values could result in different gonadal dysfunction [53], which could also contribute to the varied serum concentration of gonadal hormones in our results. In addition, elevated parathormone was found to be related to lower values of FSH, LH and higher values of PRL [54]. Therefore, the parathormone might also be an interference factor.

## Conclusion

There were consistently elevated PRL, follicular LH and FSH levels and decreased P levels, with serum levels of E_2_ varying from low to normal in HD women compared with normal control. There was also higher PRL values in HD women compared with that of women after RT.

### Strengths

This was the first meta-analysis to present the gonadal hormone and PRL values of HD women, which exhibited the function of reproductive system of HD women to some extent and gave us clues for risks estimation of cardiovascular disease, survival and so on. In addition, we did a thoroughly literature searches and took measures to adjust small-study effects to bolster the conclusion validity.

### Weaknesses

The relatively small sample size and limited information from original literature to explore and conform the source of heterogeneity were limitations. What’s more, the PROSPERO number was post-registered which might introduce some biases into the study.

### Electronic supplementary material

Below is the link to the electronic supplementary material.

Supplementary Material 1

Supplementary Material 2

Supplementary Material 3

## Data Availability

Data and materials are available by contacting the corresponding author.

## Declarations

### Founding

No funds, grants, or other support was received.
