# Supplementary material for: Serum prolactin and gonadal hormones in hemodialysis women: a meta-analysis
Source: BMC Endocr Disord. 2023 Sep 25;23:203. doi: 10.1186/s12902-023-01452-w (PMC10518945; doi:10.1186/s12902-023-01452-w)

**Supplemental Figure 1 (SFigure1).** The sensitivity analysis of prolactin values between hemodialysis women and healthy control. Every transverse line means the estimated effect (the circle in the middle of line) and its 95% confidence interval of included studies after omitting the study at the left of line.

**
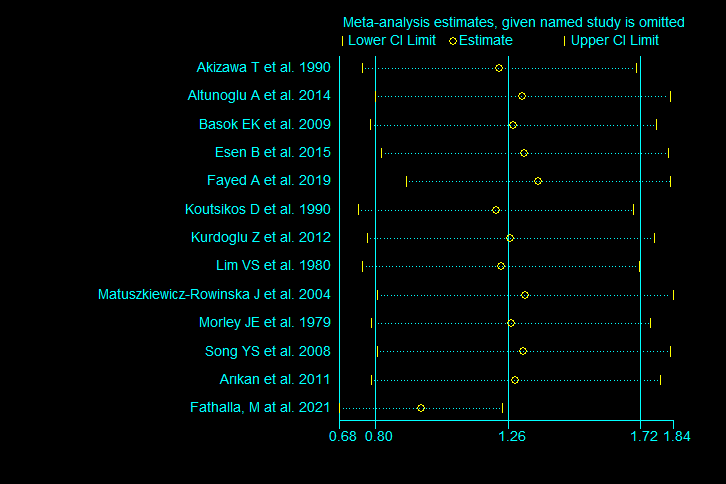
**

**Supplemental Figure 2 (SFigure2).** The metaregression analysis of prolactin values and mean age of hemodialysis women of per study in the comparison of hemodialysis women and healthy control after excluding the study of Lim VS et al. for unavailable to obtain mean age.

**
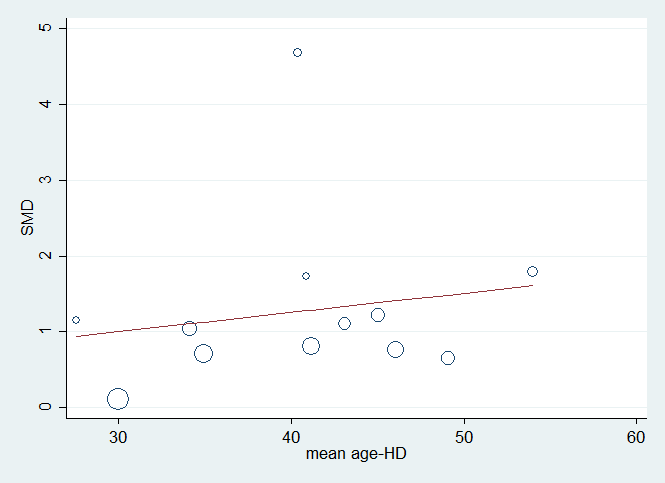
**

**Results showed there was no correlation (*p*=0.899).**

**Supplemental Figure 3 (SFigure3).** The TSA analysis of prolactin values after excluding the study of Fathalla, M at al.

**
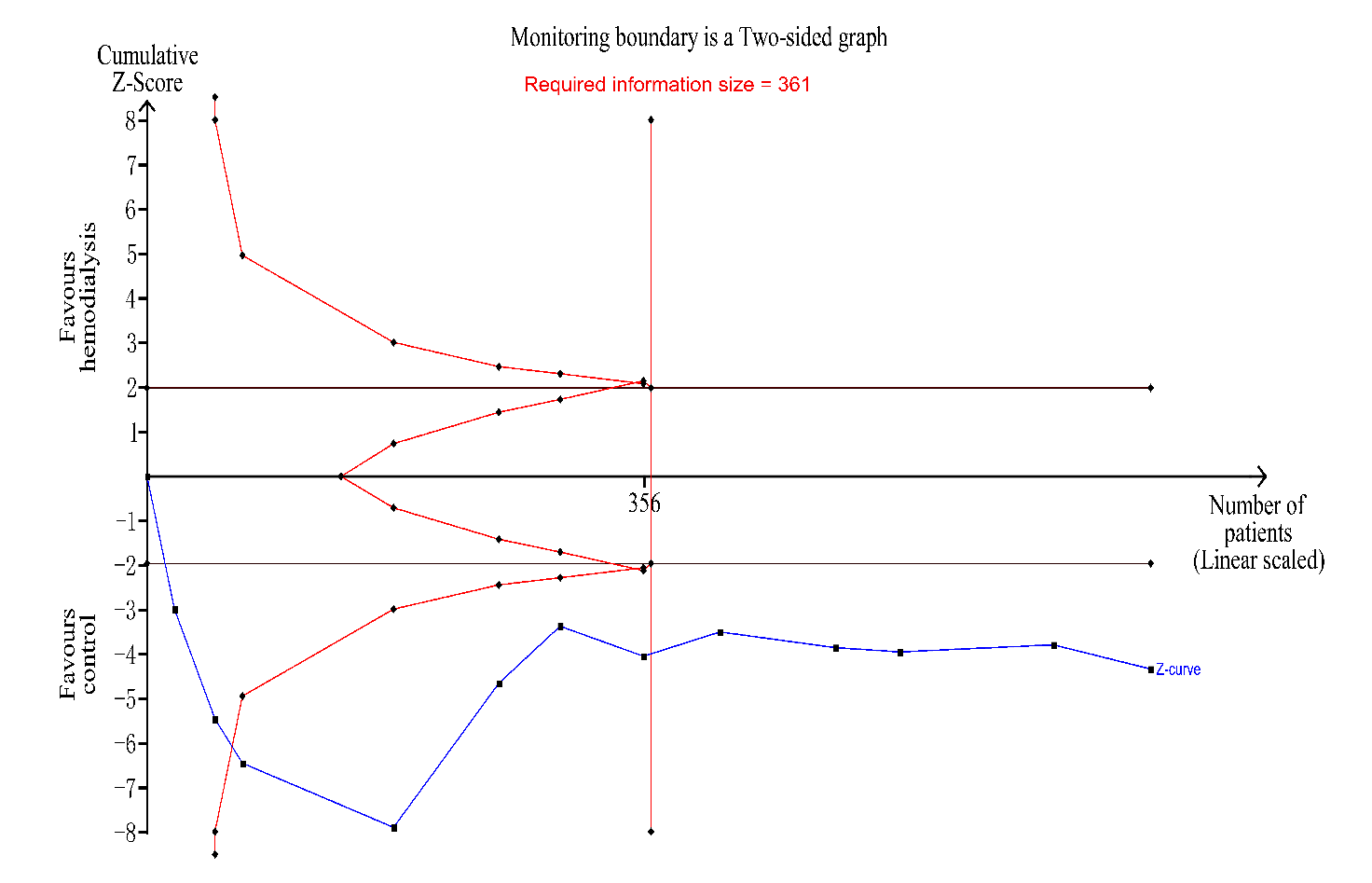
**

The Z curve stayed out of futility borders, monitoring boundaries and reached the required information size meaning that there existed enough confidence to concluded that serum PRL was higher in hemodialysis women.

**Supplemental Figure 4 (SFigure4).** The forest plot of follicular stimulating hormone (FSH) values between hemodialysis women and healthy control of all included studies. The study of Koutsikos D et al. contributed lots of heterogeneity in the subgroup of follicular phase.

**
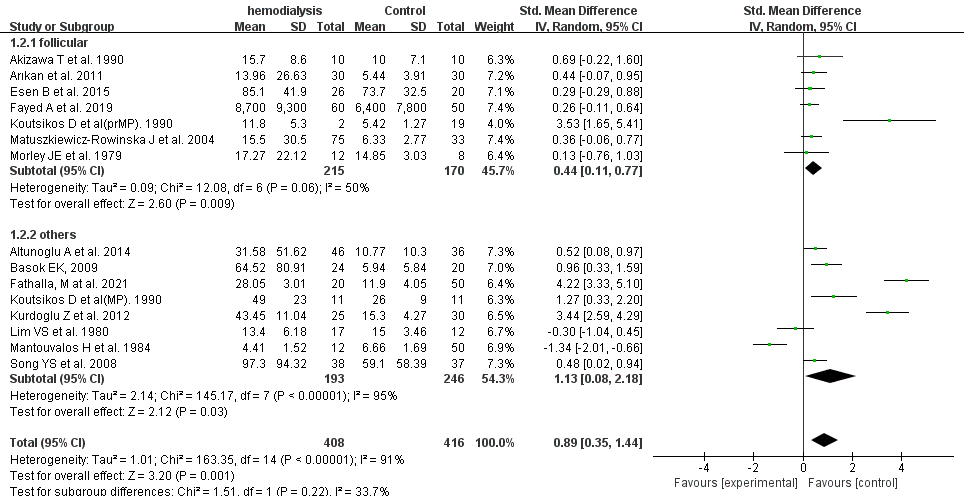
**

**Supplemental Figure 5 (SFigure5).** The sensitivity analysis of follicular stimulating hormone (FSH) values between hemodialysis women and healthy control of all studies. Every transverse line means the estimated effect (the circle in the middle of line) and its 95% confidence interval of included studies after omitting the study at the left of line.

**
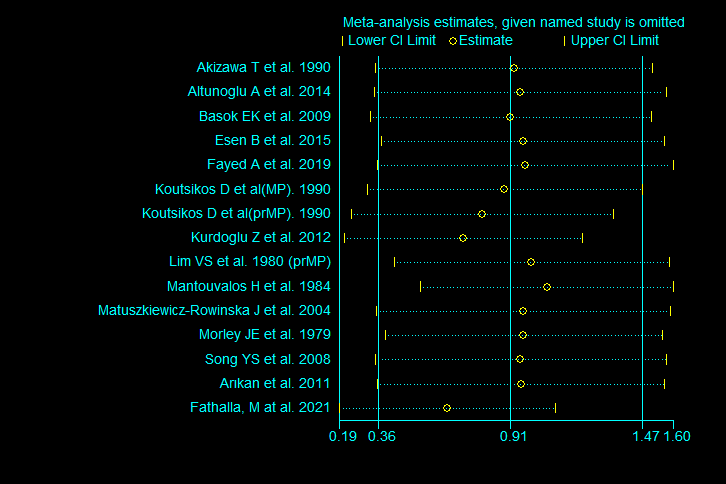
**

**Supplemental Figure 6 (SFigure 6).** The metaregression analysis of FSH values and mean age of hemodialysis women of per study in the comparison of hemodialysis women and healthy control.

**
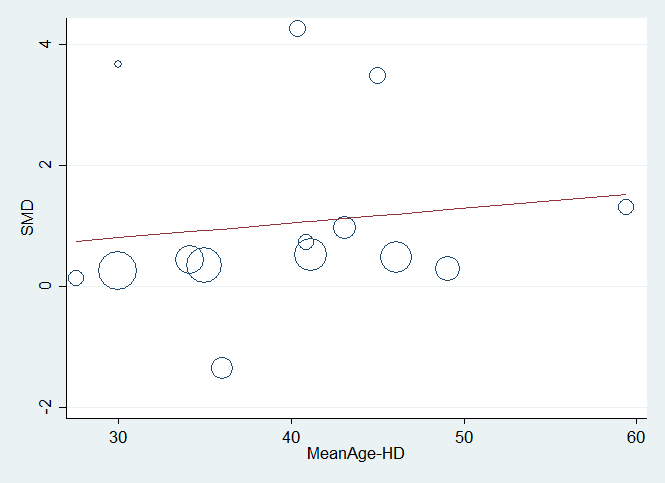
**

**Results showed there was no correlation (*p*=0.974).**

**Supplemental Figure 7 (SFigure 7).** The TSA analysis of follicular stimulating hormone (FSH) of all included studies in HD vs. CT group and the Z curve finally stayed inner the conventional boundary meaning a further study was needed to confirm the conclusion.

**
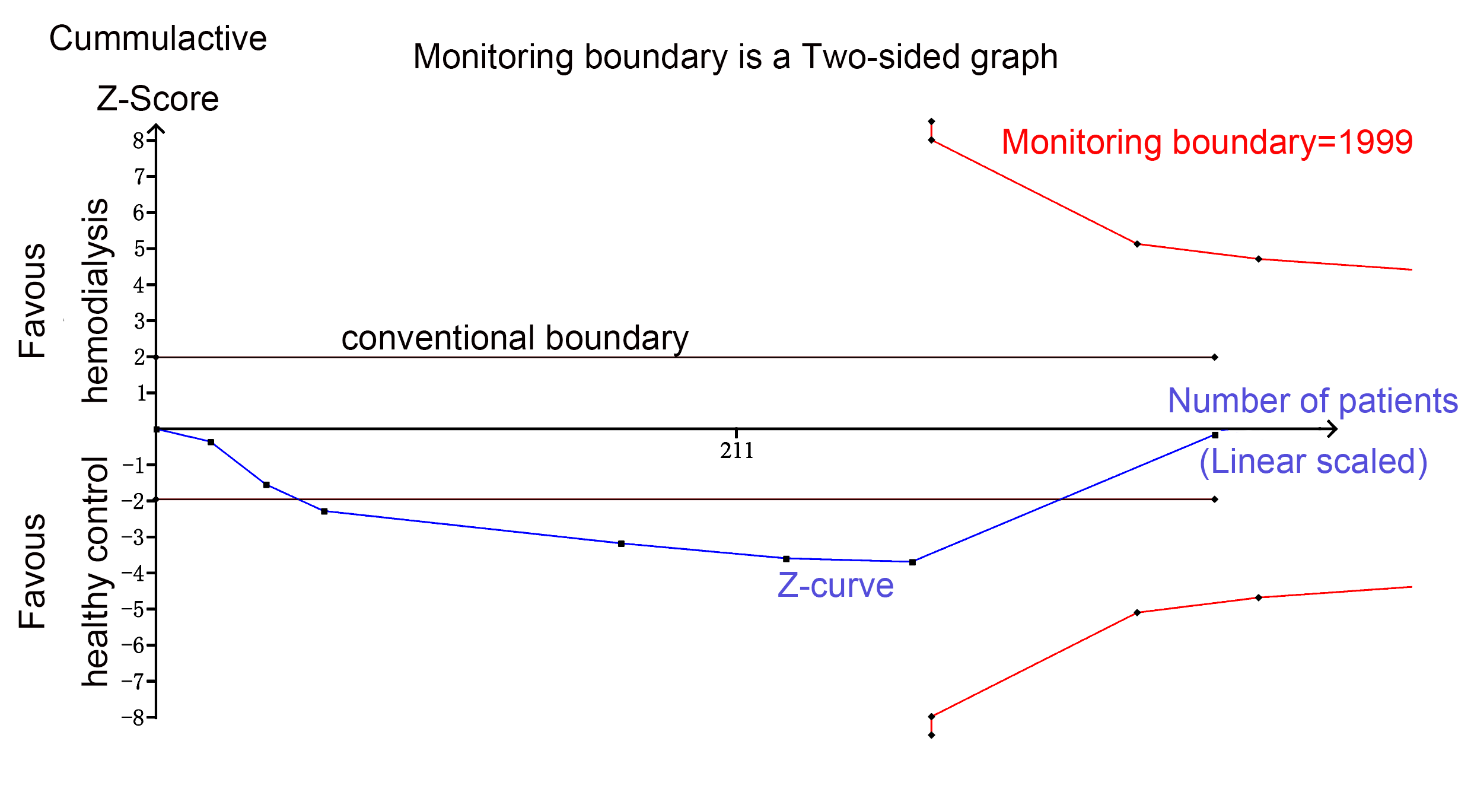
**

**Supplemental Figure 8 (SFigure 8).** The forest plot of luteinizing hormone (LH) values between hemodialysis women and healthy control of all included studies. The study of Koutsikos D et al (Koutsikos D et al.(prMP)) showed largre heterogeneity.

**
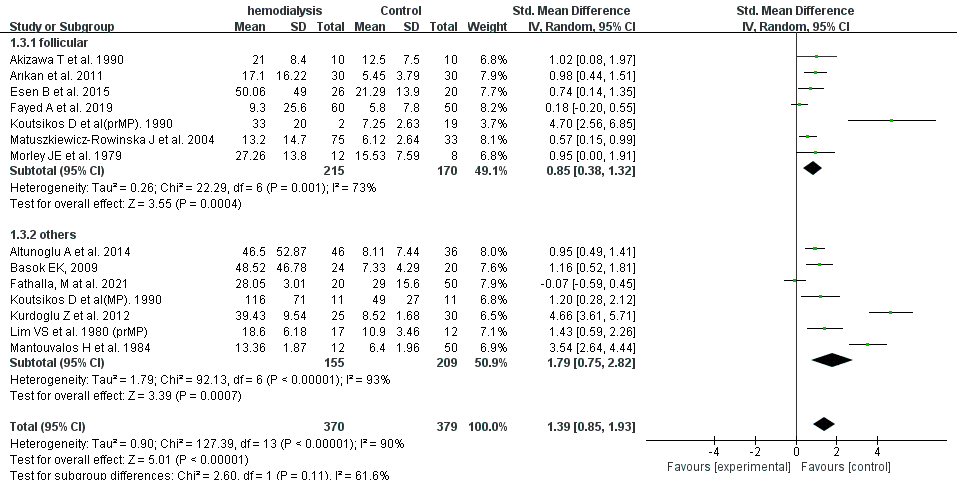
**

**Supplemental Figure 9 (SFigure 9).** The sensitivity analysis of luteinizing hormone (LH) values between hemodialysis women and healthy control of all studies. Every transverse line means the estimated effect (the circle in the middle of line) and its 95% confidence interval of included studies after omitting the study at the left of line.

**
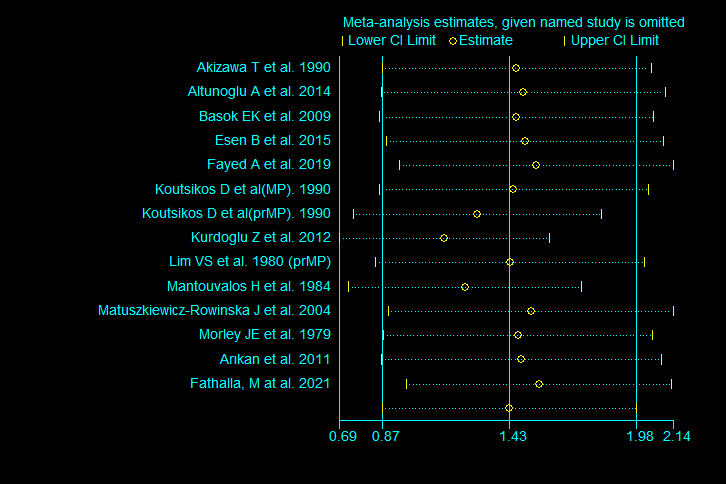
**

**Supplemental Figure 10 (SFigure 10).** The metaregression analysis of LH values and mean age of hemodialysis women of per study in the comparison of hemodialysis women and healthy control.

**
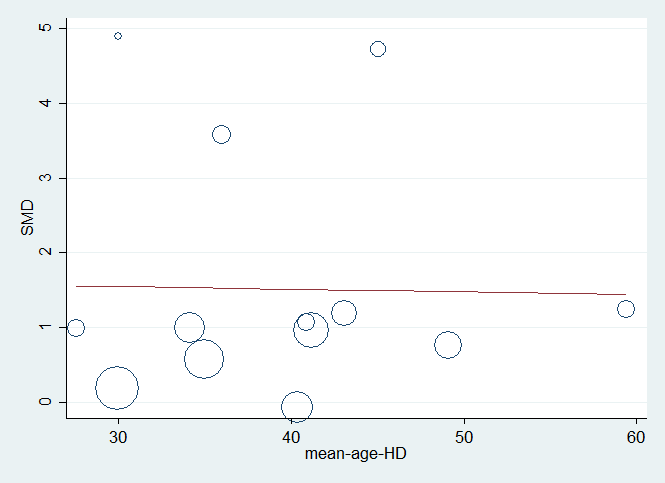
**

**Results showed there was no correlation (*p*=0.471).**

**Supplemental Figure 11 (SFigure11).** The forest plot of E_2_ values between hemodialysis women and healthy control after excluding the study of Koutsikos D et al. (Koutsikos D et al.(prMP)) showing higher heterogeneity.


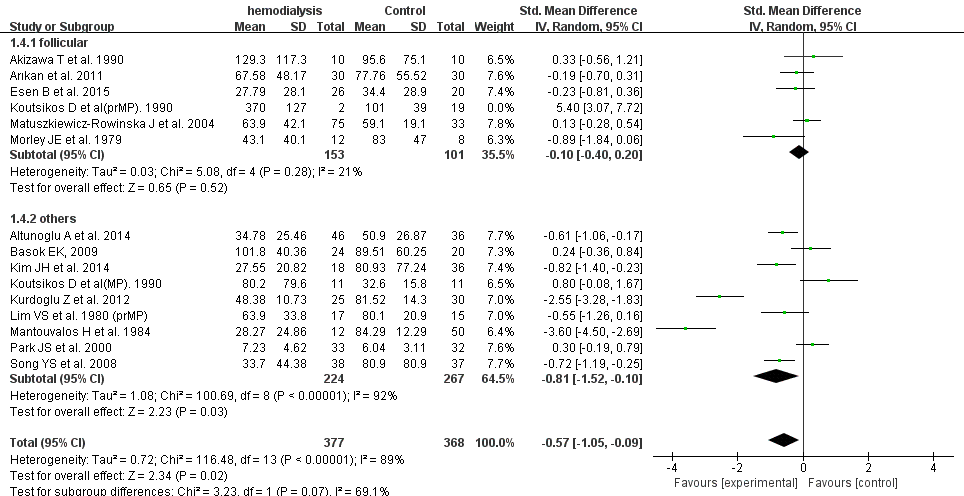


**Supplemental Figure 12 (SFigure12).** The forest plot of E_2_ values between hemodialysis women and healthy control of all included studies.


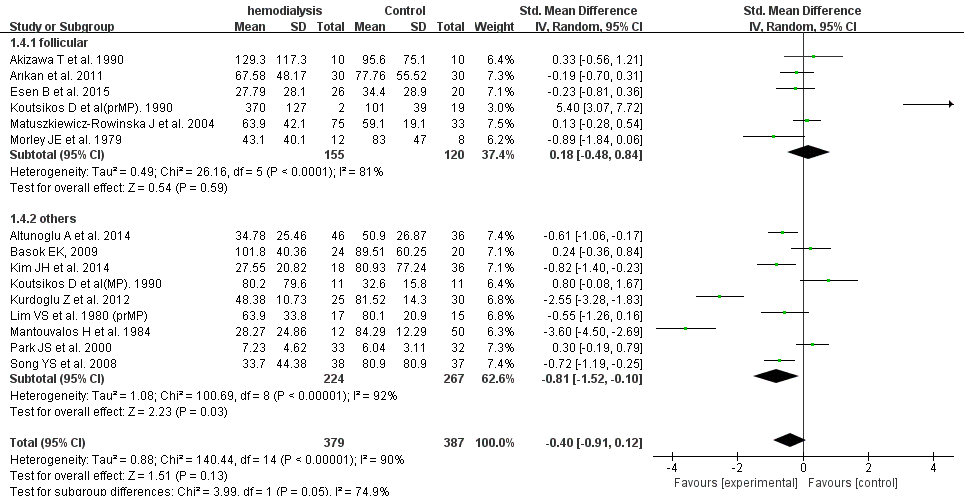


**Supplemental Figure 13 (SFigure 13).** The sensitivity analysis of E_2_ values between hemodialysis women and healthy control of all studies. Every transverse line means the estimated effect (the circle in the middle of line) and its 95% confidence interval of included studies after omitting the study at the left of line.


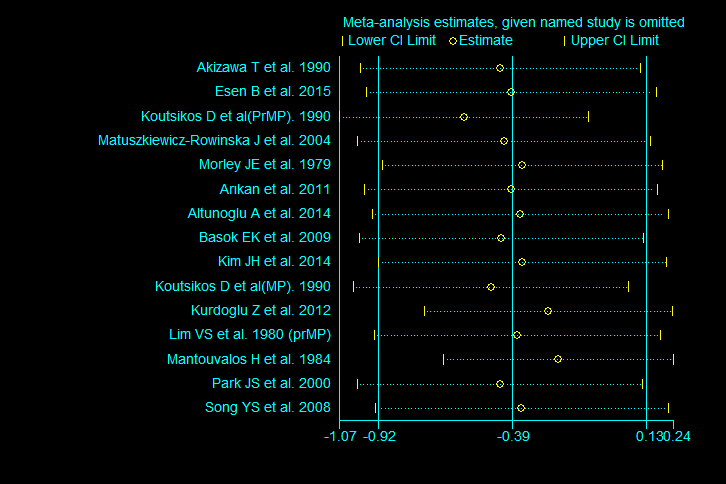


**Supplemental Figure 14 (SFigure 14).** The metaregression analysis of E_2_ values and mean age of hemodialysis women of per study in the comparison of hemodialysis women and healthy control after excluding the study of Lim VS et al. unable to get the mean age.


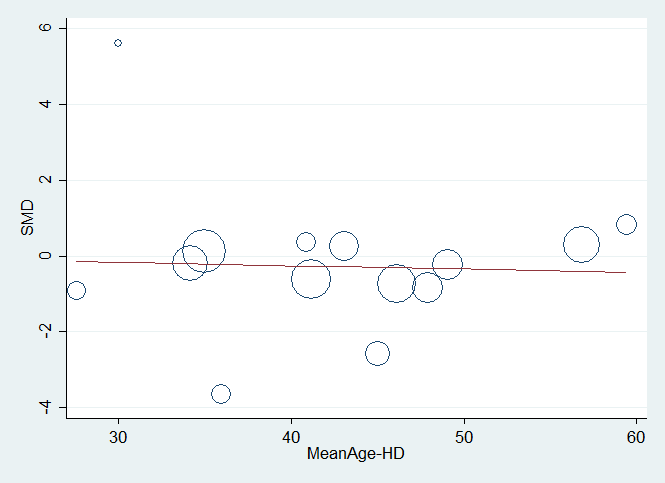


**Results showed there was no correlation (*p*=0.970).**

**Supplemental Figure 15 (SFigure 15).** The TSA analysis of E_2_ of all included studies in HD vs. CT group and the Z curve stayed within the conventional boundary and the required information size boundary was ignored in the graph due to not enough of studies. A, TSA analysis of all included studies; B, TSA analysis of E_2_ tested during follicular phase.


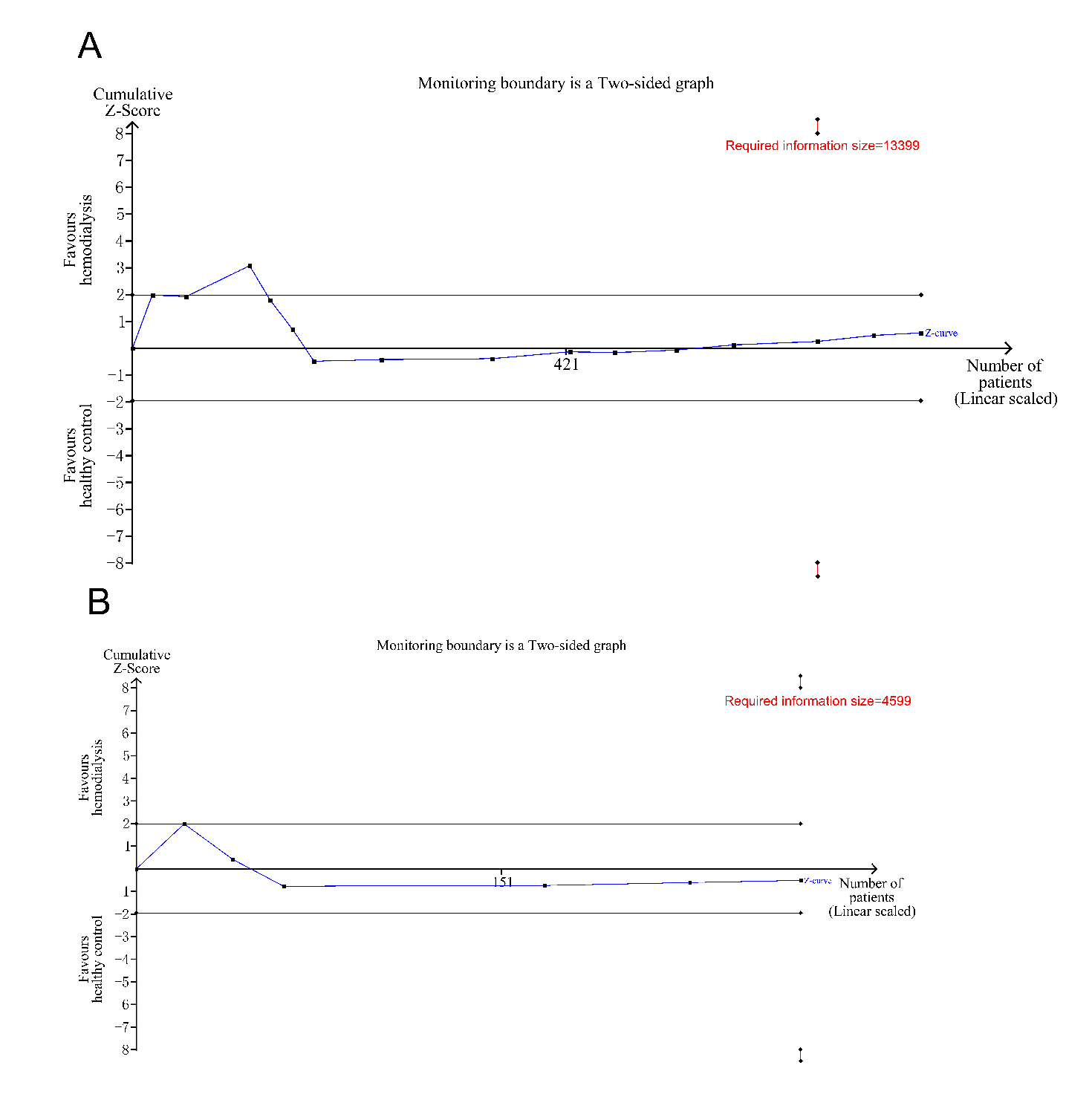


**Supplemental Figure 16 (SFigure 16).** The sensitivity analysis of progestone values between hemodialysis women and healthy control. Every transverse line means the estimated effect (the circle in the middle of line) and its 95% confidence interval of included studies after omitting the study at the left of line.


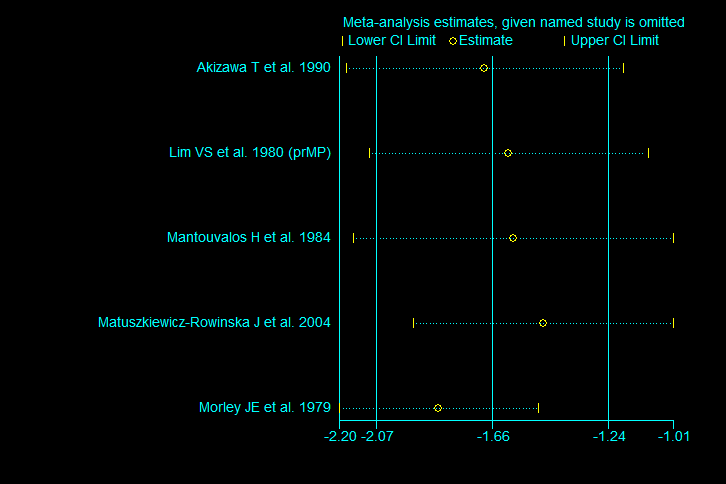


**Supplemental Figure 17 (SFigure 17).** The sensitivity analysis of PRL values between hemodialysis women and women after renal transplantation. Every transverse line means the estimated effect (the circle in the middle of line) and its 95% confidence interval of included studies after omitting the study at the left of line.


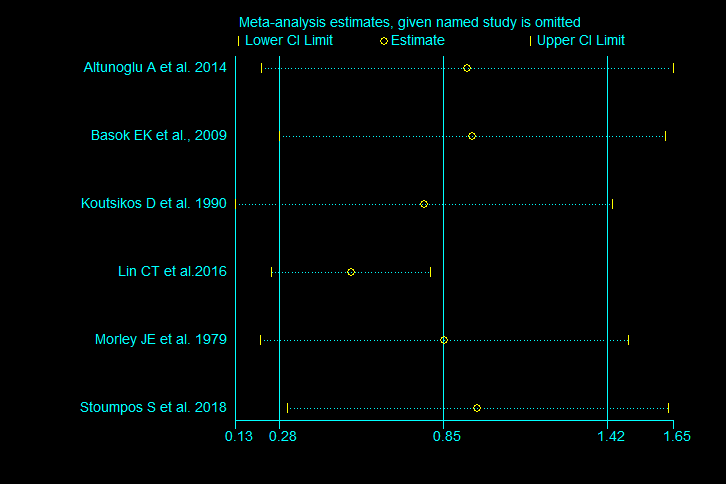


**Supplemental Figure 18 (SFigure 18).** The forest plot of FSH values between hemodialysis women and women after renal transplantation.


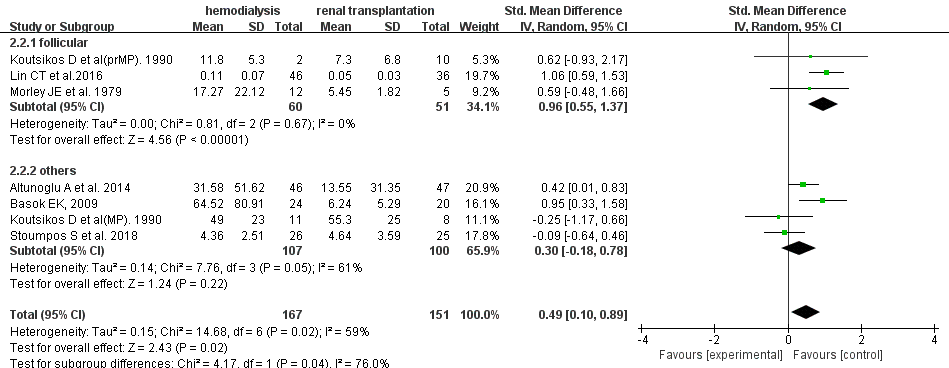


**Supplemental Figure 19 (SFigure 19).** The sensitivity analysis of FSH values between hemodialysis women and women after renal transplantation. Every transverse line means the estimated effect (the circle in the middle of line) and its 95% confidence interval of included studies after omitting the study at the left of line.


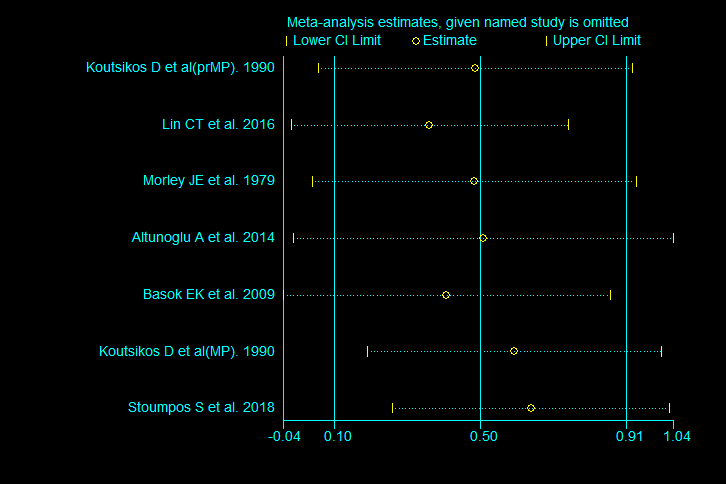


**Supplemental Figure 20 (SFigure 20).** The TSA analysis of FSH of all included studies in HD vs. RT group and the monitoring boundary was ignored due to not enough studies included.


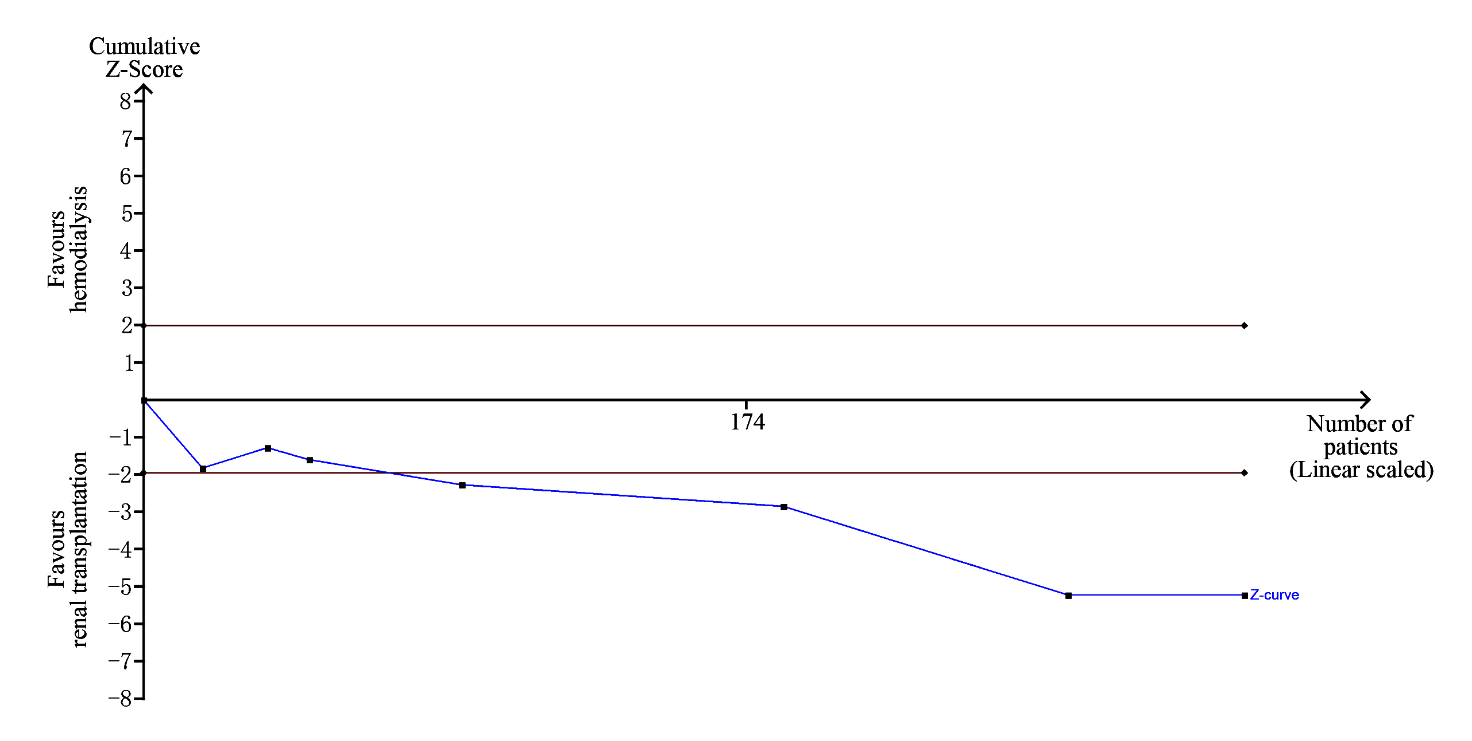


**Supplemental Figure 21 (SFigure 21).** The forest plot of LH values between hemodialysis women and women after renal transplantation.


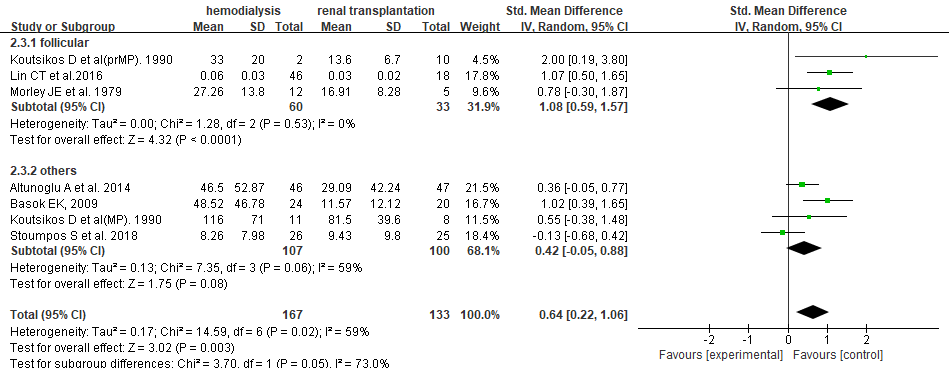


**Supplemental Figure 22 (SFigure 22).** The sensitivity analysis of LH values between hemodialysis women and women after renal transplantation (HD vs. RT). Every transverse line means the estimated effect (the circle in the middle of line) and its 95% confidence interval of included studies after omitting the study at the left of line.


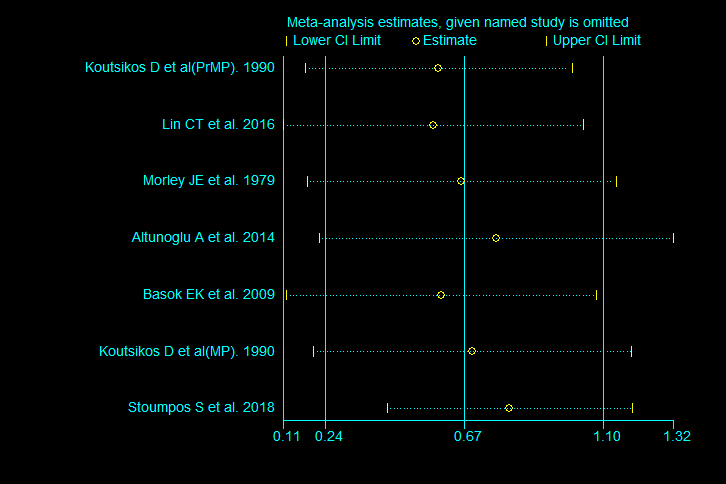


**Supplemental Figure 23 (SFigure 23).** The TSA analysis of LH of all included studies in HD vs. RT group and the Z curve didn’t reach the monitoring boundary, the futility boundary and the required information size.


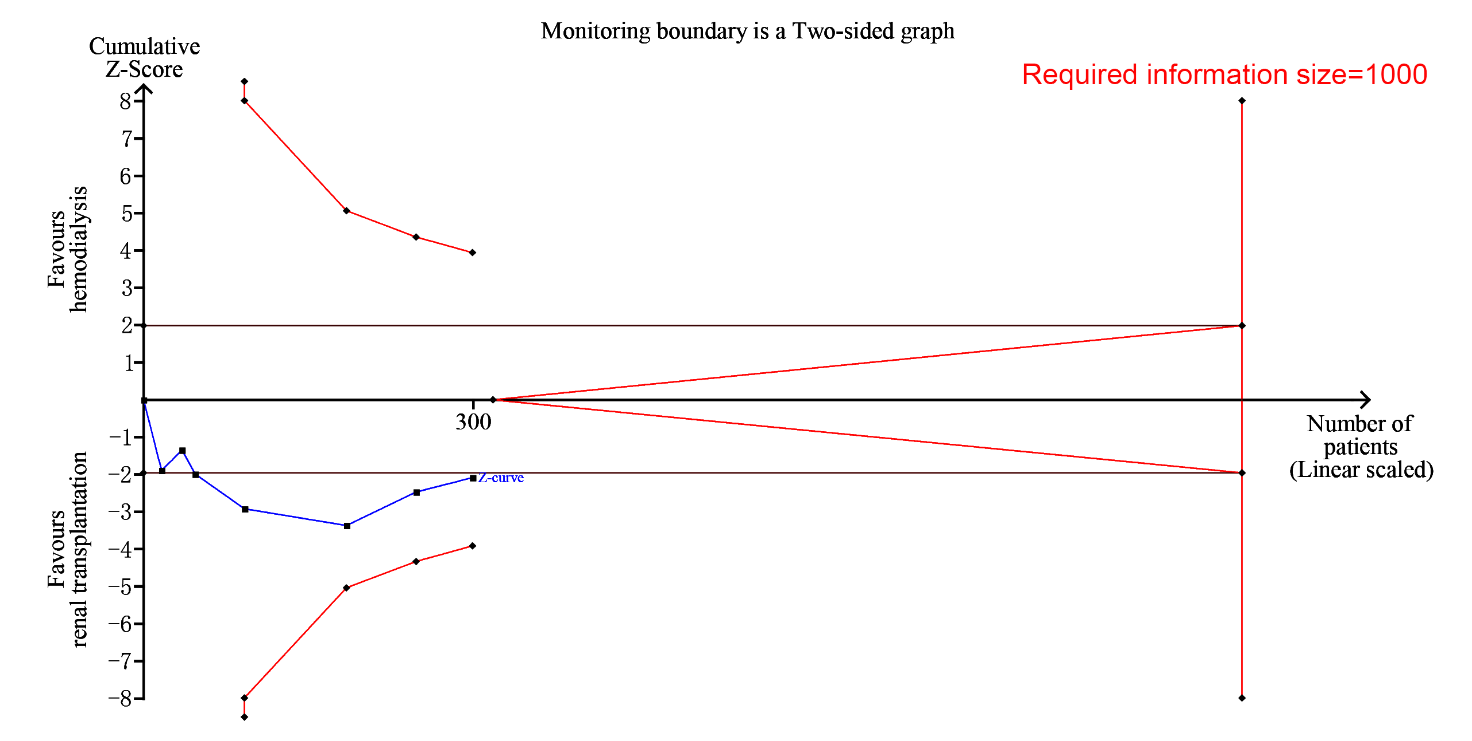


**Supplemental Figure 24 (SFigure24).** The forest plot of E_2_ values between hemodialysis women and women after renal transplantation.


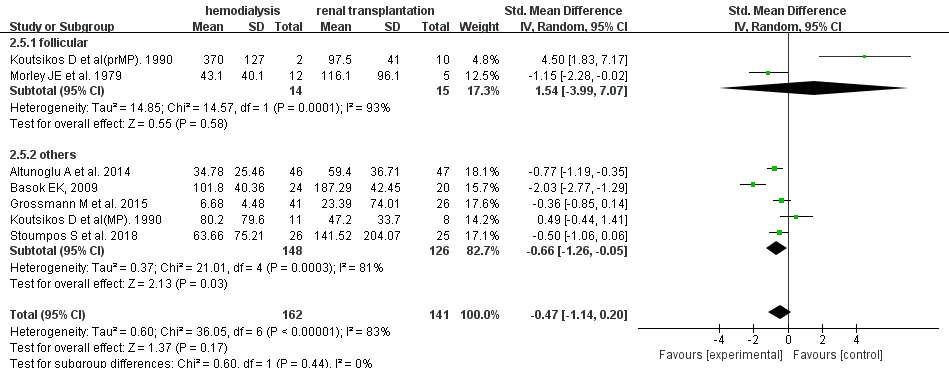


**Supplemental Figure 25 (SFigure 25).** The sensitivity analysis of E_2_ values between hemodialysis women and women after renal transplantation (HD vs. RT). Every transverse line means the estimated effect (the circle in the middle of line) and its 95% confidence interval of included studies after omitting the study at the left of line.


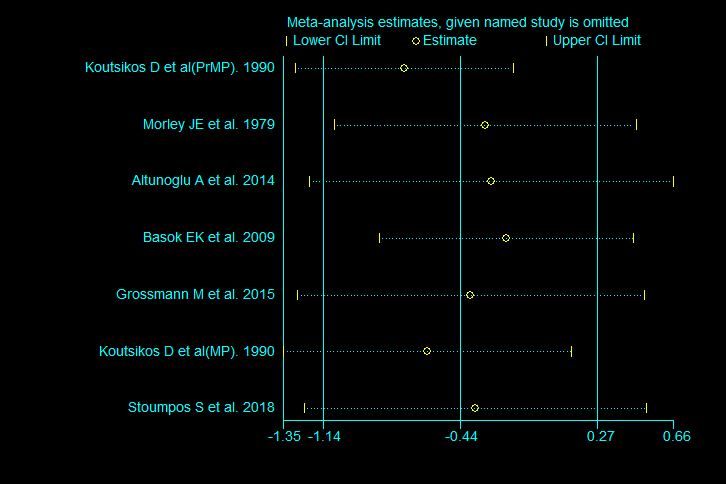


**Supplemental Figure 26 (SFigure 26).** The TSA analysis of E_2_ values of all included studies in HD vs. RT group and the monitoring boundary was ignored because of not enough studies included.


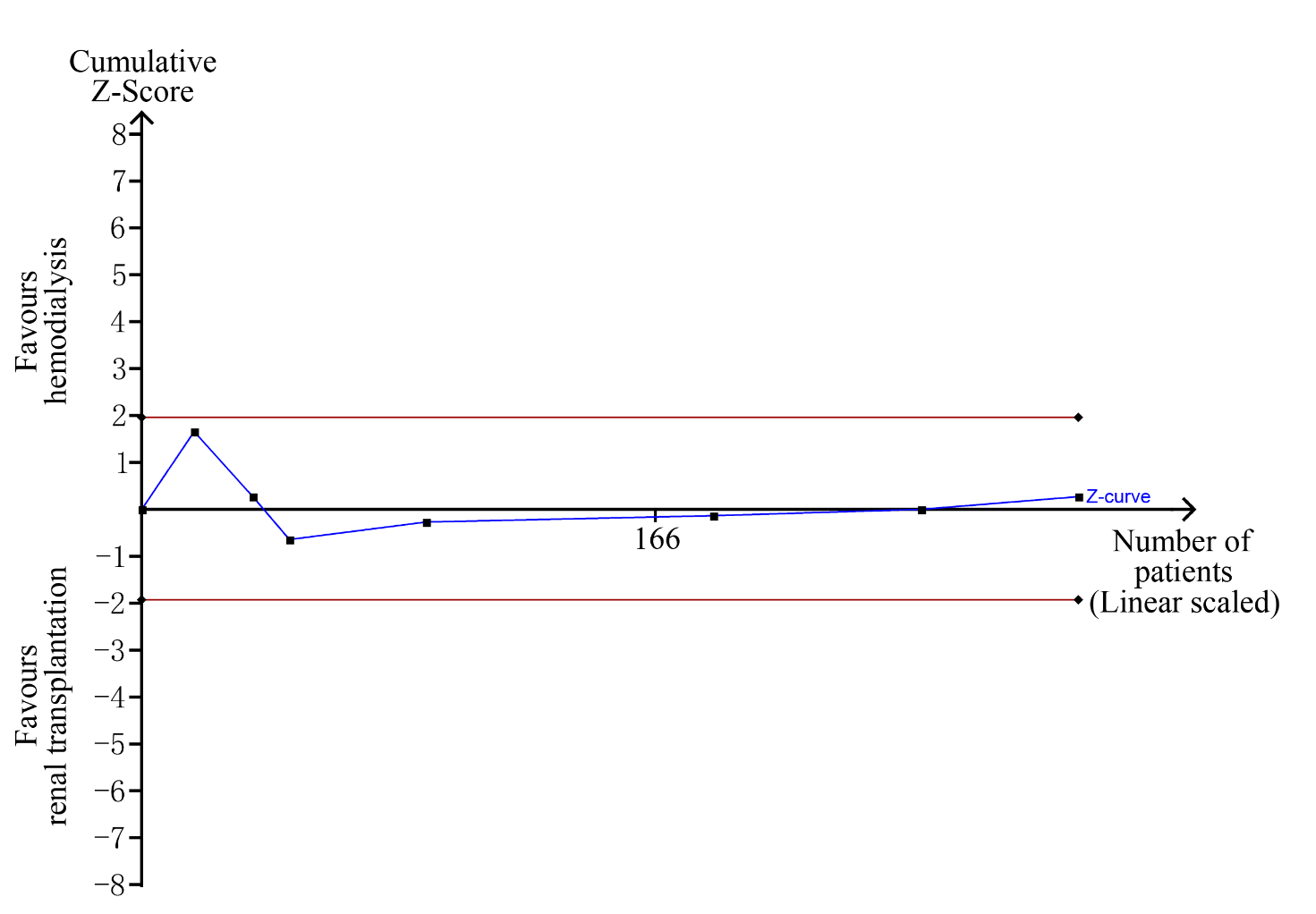


**Supplemental Figure 27 (SFigure 27).** The forest plot of P values between hemodialysis women and women after renal transplantation.


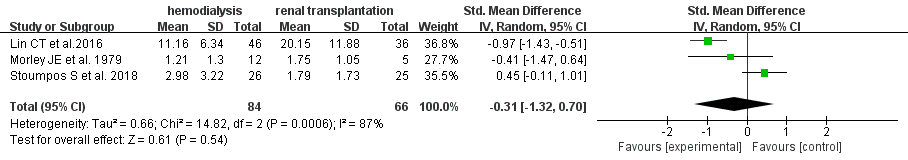


**Supplemental Figure 28 (SFigure 28).** The TSA analysis of P values of all included studies in HD vs. RT group and the Z curve didn’t reach the monitoring boundary, the futility boundary and the required information size.


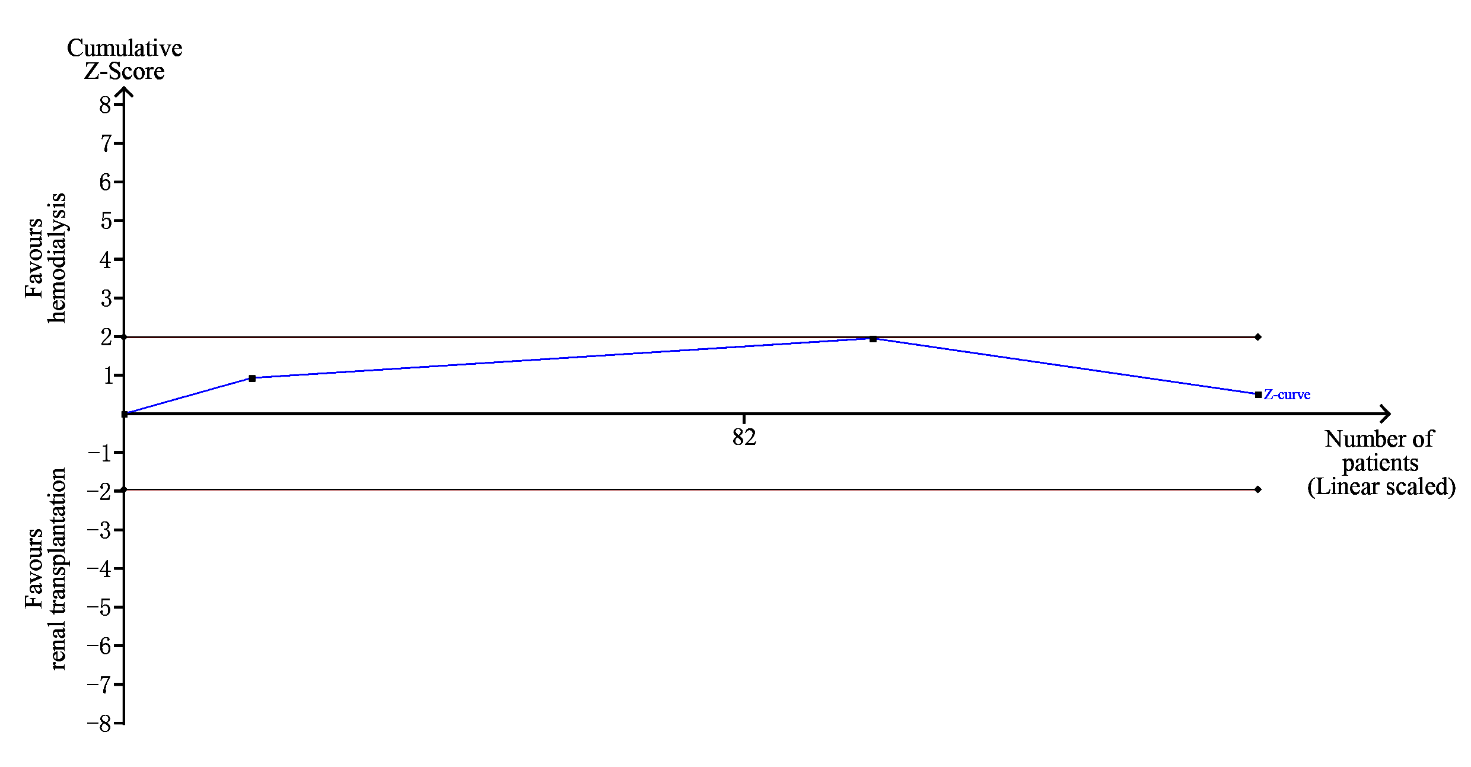


**Supplemental Figure 29 (SFigure 29).** The forest plot of PRL values between regular hemodialysis women and irregular hemodialysis women (HDre vs. HDir).


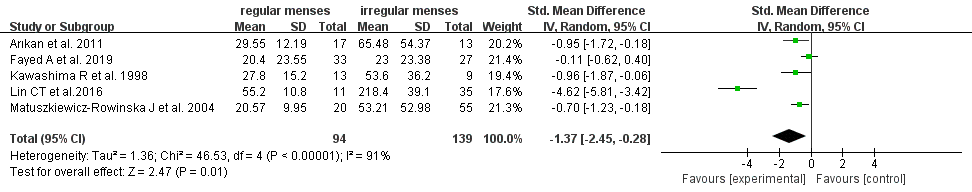


**Supplemental Figure 30 (SFigure 30).** The forest plot of PRL values between regular hemodialysis women and irregular hemodialysis women (HDre vs. HDir) after excluding the study of Lin CT et al.


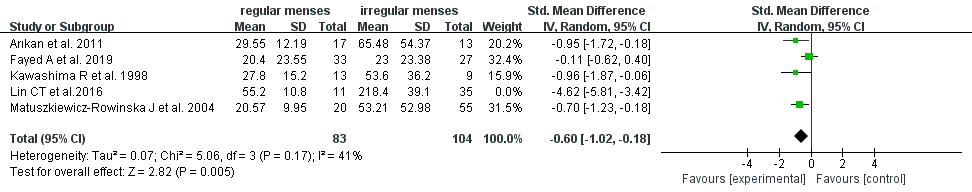


**Supplemental Figure 31 (SFigure 31).** The sensitivity analysis of PRL values in HDre vs. HDir group. Every transverse line means the estimated effect (the circle in the middle of line) and its 95% confidence interval of included studies after omitting the study at the left of line.


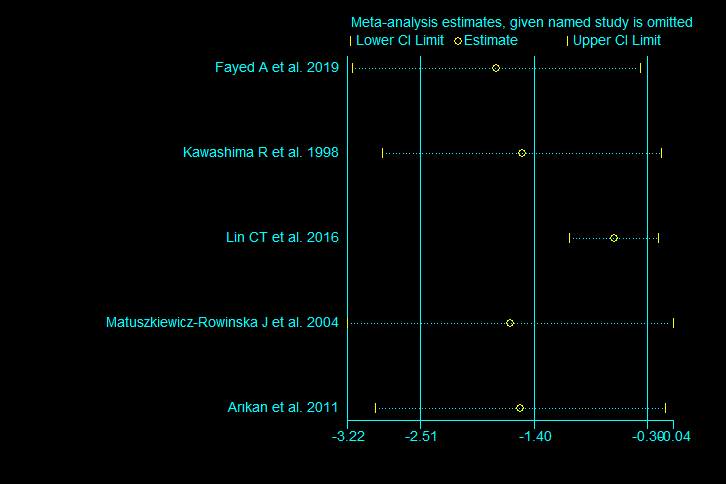


**Supplemental Figure 32 (SFigure 32).** The TSA analysis of PRL values of all included studies in HDre vs. HDir group and the Z curve didn’t reach the monitoring boundary, the futility boundary and the required information size.


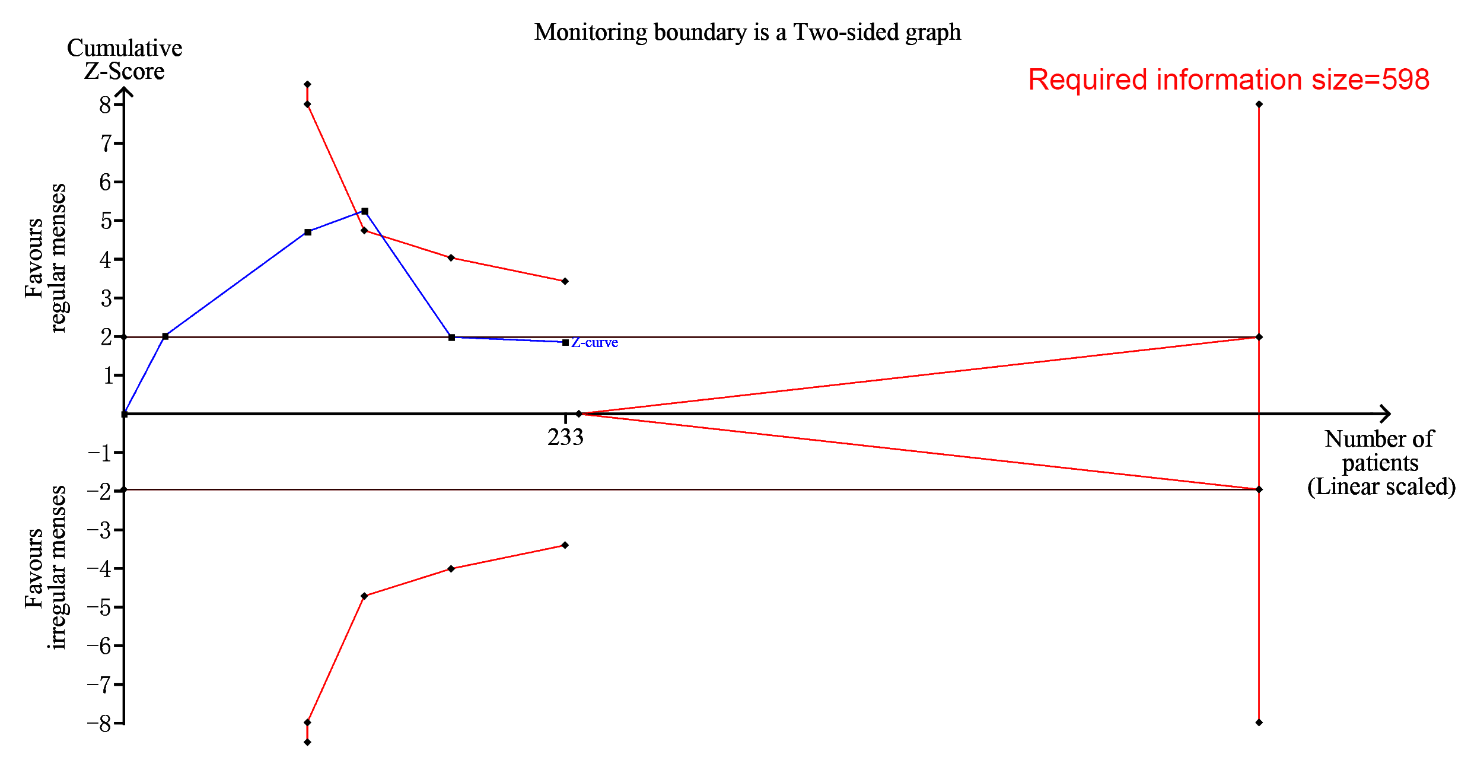


**Supplemental Figure 33 (SFigure 33).** The forest plot of FSH values between regular hemodialysis women and irregular hemodialysis women (HDre vs. HDir).


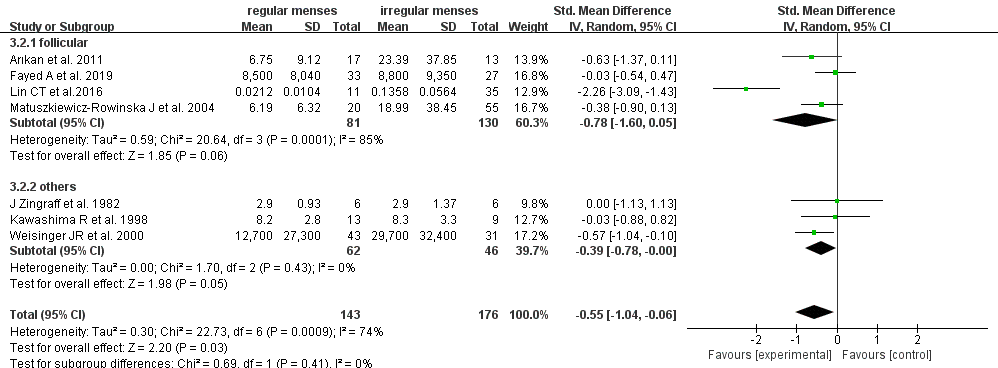


**Supplemental Figure 34 (SFigure 34).** The sensitivity analysis of FSH values in HDre vs. HDir group. Every transverse line means the estimated effect (the circle in the middle of line) and its 95% confidence interval of included studies after omitting the study at the left of line.


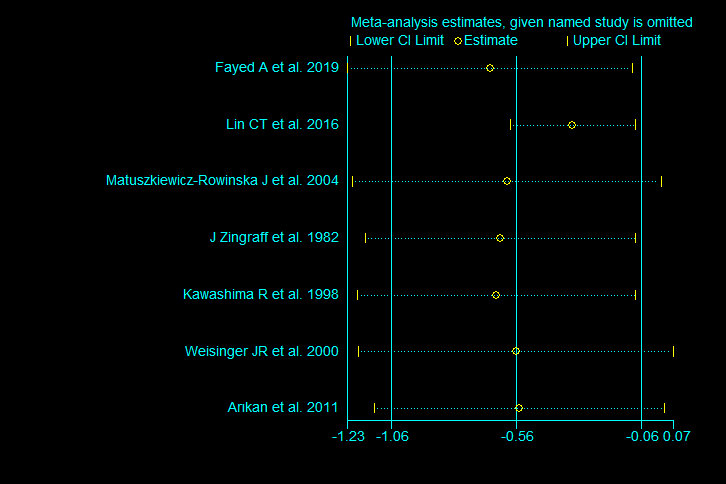


**Supplemental Figure 35 (SFigure 35).** The forest plot of FSH values between regular hemodialysis women and irregular hemodialysis women (HDre vs. HDir) after excluding the study of Lin CT at al.


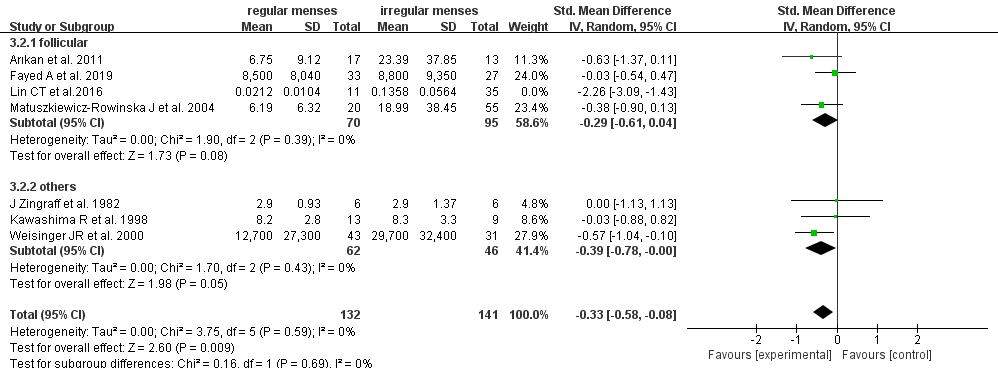


**Supplemental Figure 36 (SFigure 36).** The TSA analysis of FSH values of all included studies in HDre vs. HDir group and the Z curve didn’t reach the monitoring boundary, the futility boundary and the required information size. A, TSA analysis of FSH values tested during follicular phase; B, TSA analysis of FSH values in all included studies.


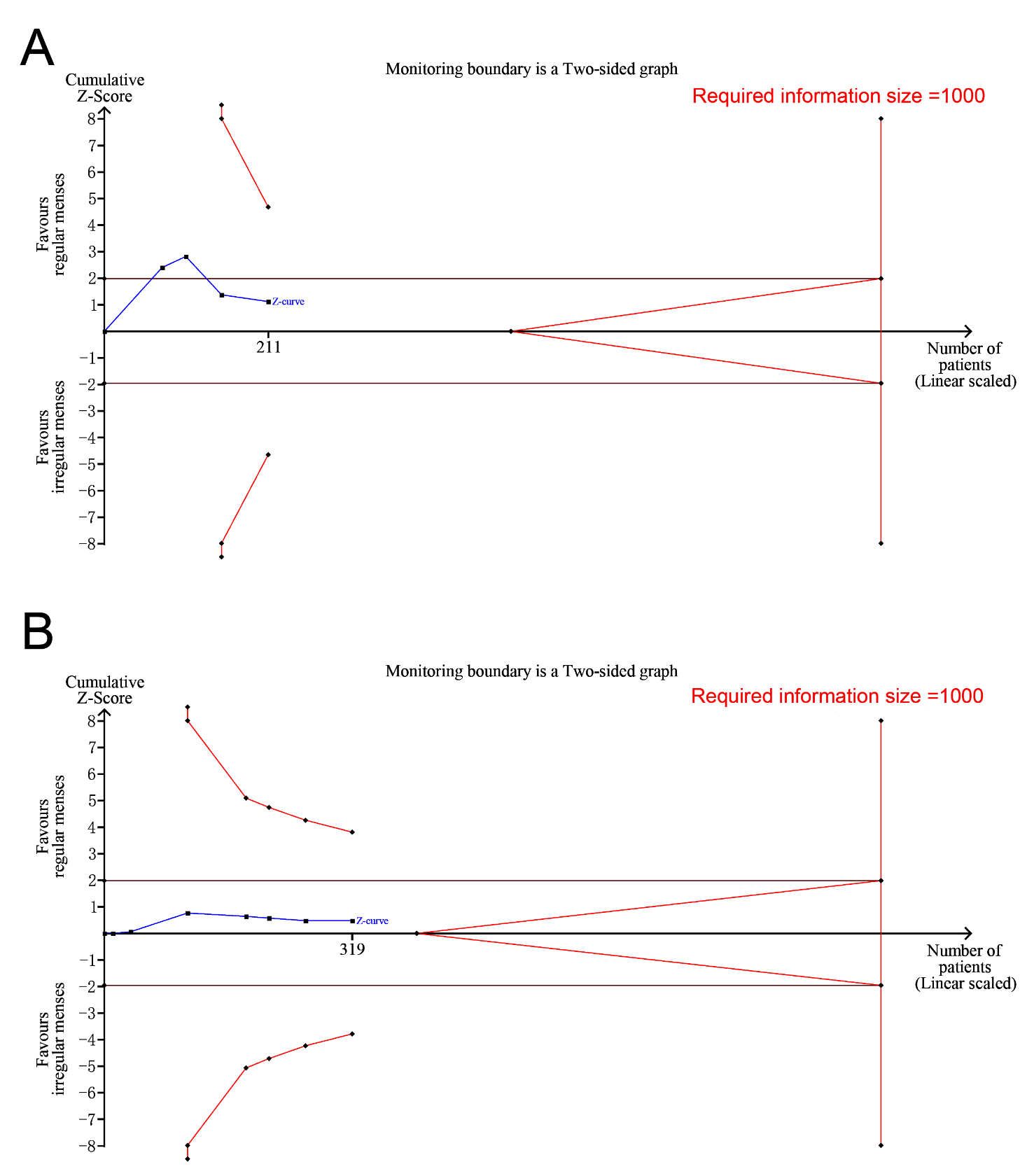


**Supplemental Figure 37 (SFigure 37).** The forest plot of LH values between regular hemodialysis women and irregular hemodialysis women (HDre vs. HDir).


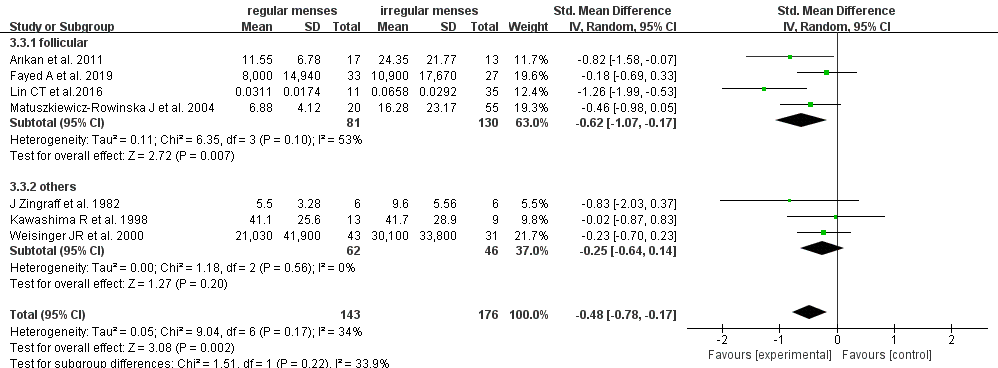


**Supplemental Figure 38 (SFigure 38).** The sensitivity analysis of LH values in HDre vs. HDir group. Every transverse line means the estimated effect (the circle in the middle of line) and its 95% confidence interval of included studies after omitting the study at the left of line.


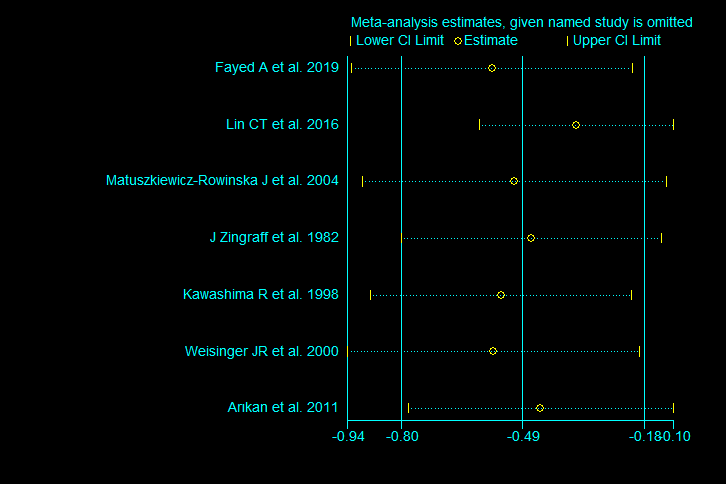


**Supplemental Figure 39 (SFigure 39).** The TSA analysis of LH values of all included studies in HDre vs. HDir group and the Z curve didn’t reach the monitoring boundary, the futility boundary and the required information size.


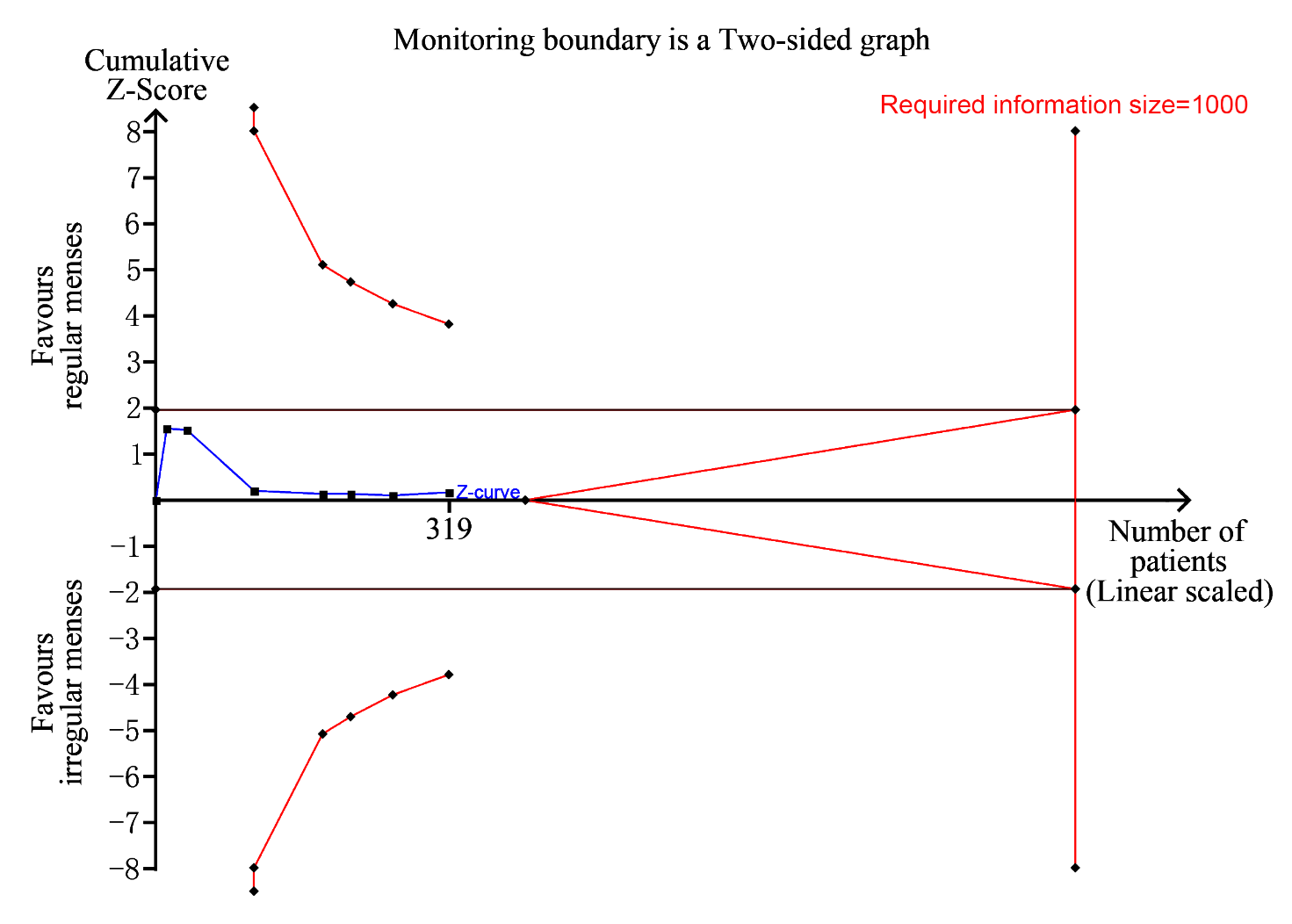


**Supplemental Figure 40 (SFigure 40).** The forest plot of E_2_ values between regular hemodialysis women and irregular hemodialysis women (HDre vs. HDir).


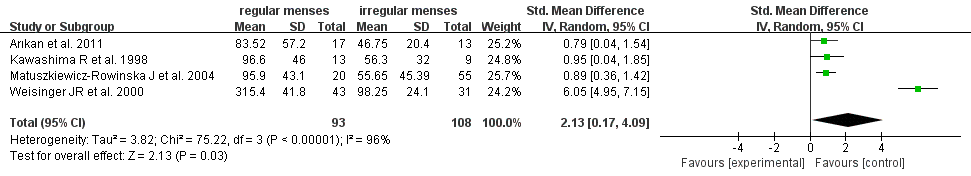


**Supplemental Figure 41 (SFigure 41).** The forest plot of E_2_ values between regular hemodialysis women and irregular hemodialysis women (HDre vs. HDir) after excluding the study of Weisinger JR et al.


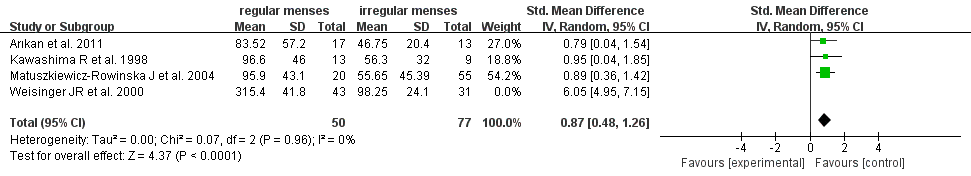


**Supplemental Figure 42 (SFigure 42).** The sensitivity analysis of E_2_ values in HDre vs. HDir group. Every transverse line means the estimated effect (the circle in the middle of line) and its 95% confidence interval of included studies after omitting the study at the left of line.


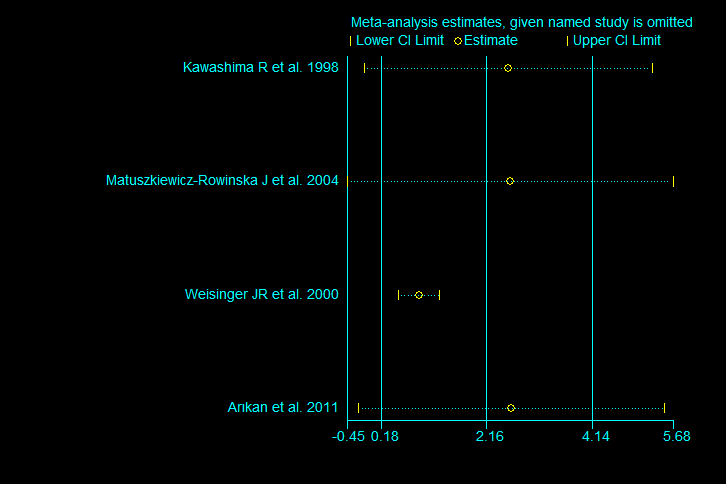


**Supplemental Figure 43 (SFigure 43).** The TSA analysis of E_2_ values between regular hemodialysis women and irregular hemodialysis women (HDre vs. HDir) and the Z curve didn’t reach the monitoring boundary, the futility boundary and the required information size.


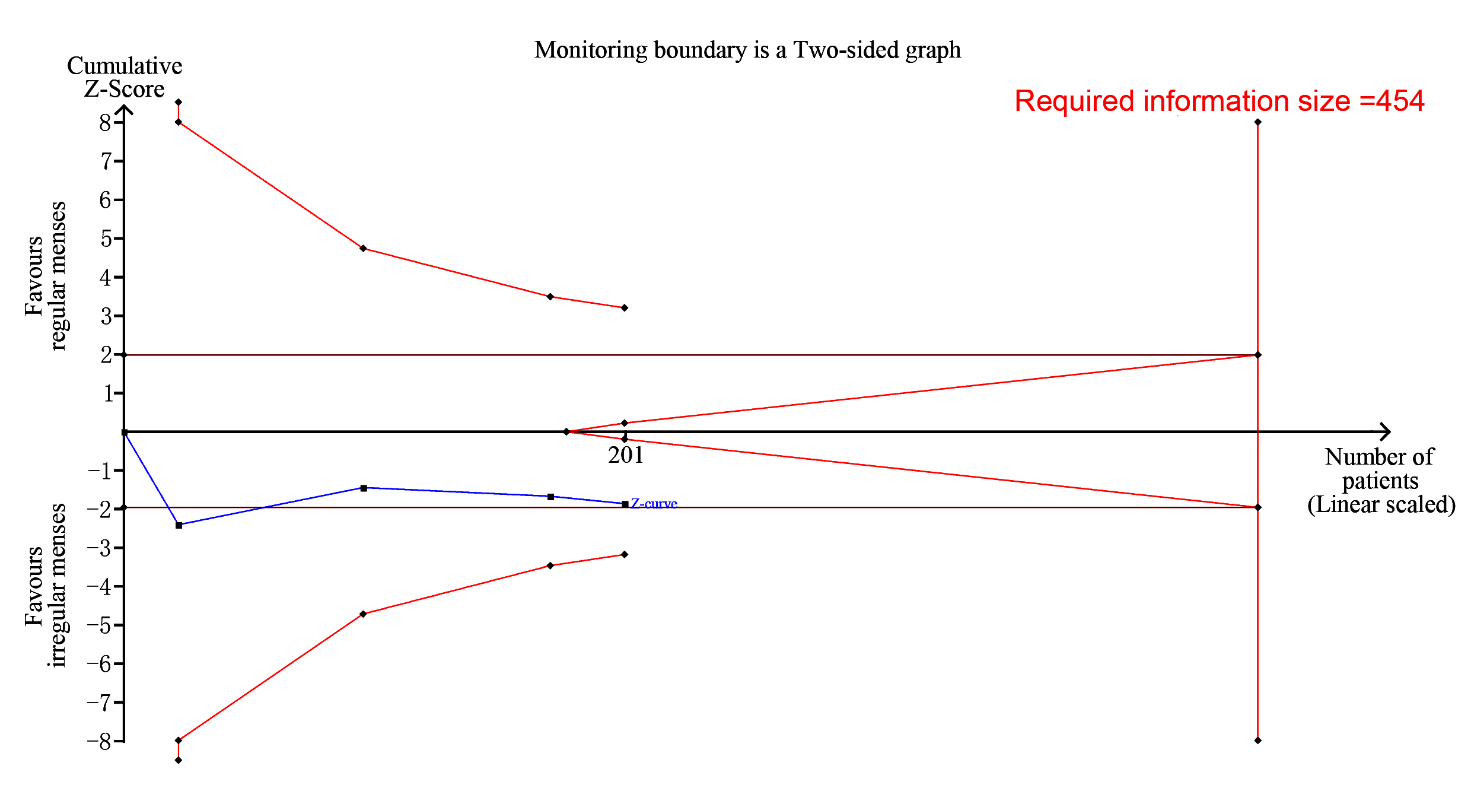


**Supplemental Figure 44 (SFigure 44).** The forest plot of P values between regular hemodialysis women and irregular hemodialysis women (HDre vs. HDir).


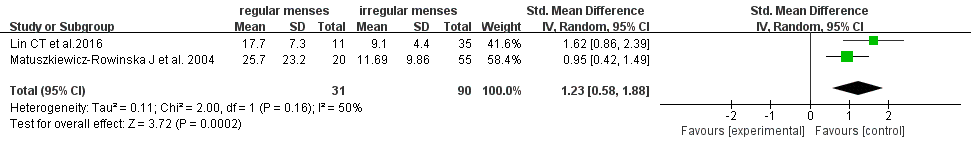


**Supplemental Figure 45 (SFigure 45).** The TSA analysis of P values of all included studies in HDre vs. HDir group. The monitoring boundary, the futility boundary and the required information size was ignored due to few studies included.


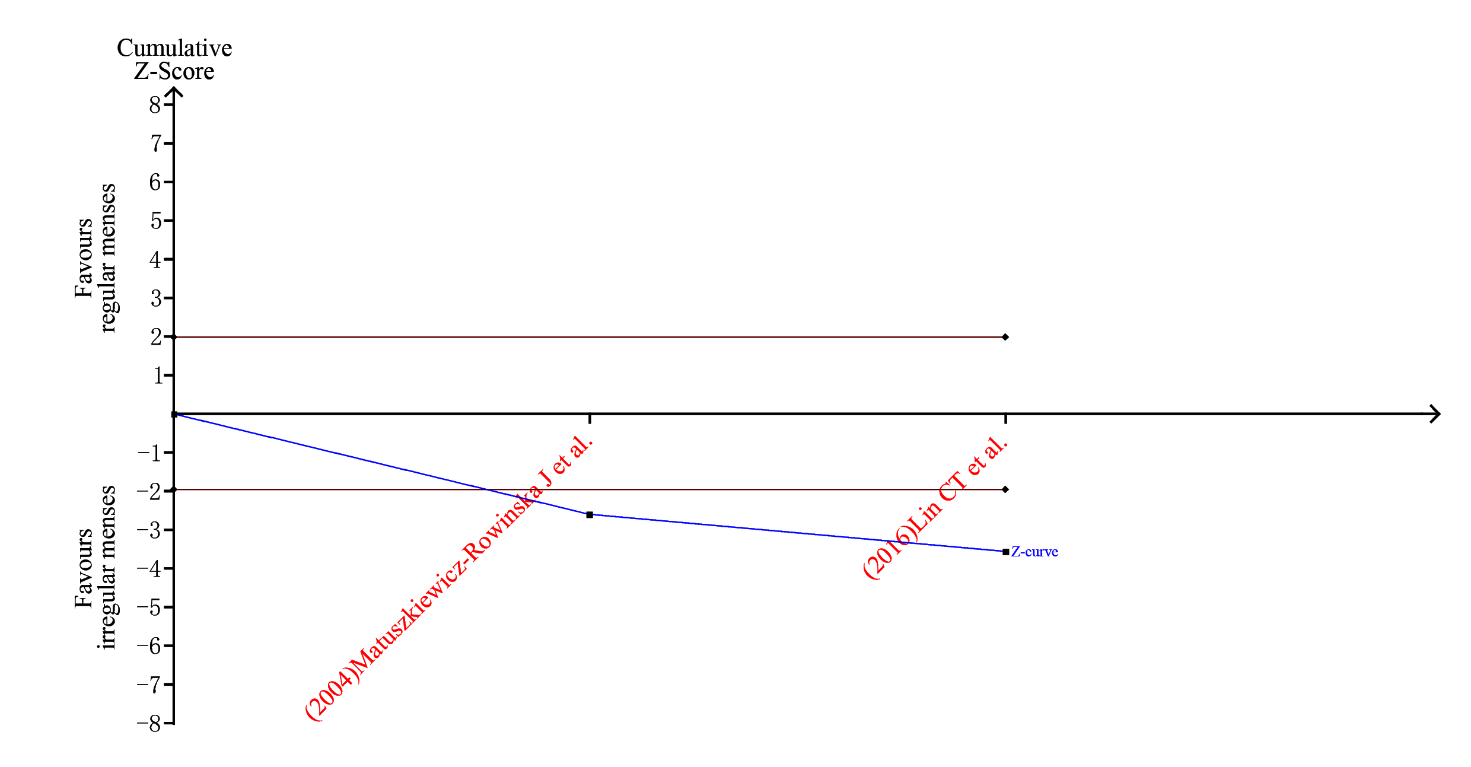

Supplement: Supplementary file 2 — Supplementary Material 2 [file 12902_2023_1452_MOESM2_ESM.docx]
