# Supplementary material for: Serum prolactin and gonadal hormones in hemodialysis women: a meta-analysis
Source: BMC Endocr Disord. 2023 Sep 25;23:203. doi: 10.1186/s12902-023-01452-w (PMC10518945; doi:10.1186/s12902-023-01452-w)
Supplement: Supplementary file 3 — Supplementary Material 3 [file 12902_2023_1452_MOESM3_ESM.docx]

**Supplemental Table 1 (STable1)**. Literature online search strategies.

| **Database** | **Search strategy** |
| --- | --- |
| PubMed | (((("gonadal hormones"[MeSH Terms] OR ("gonadal"[All Fields] AND "hormones"[All Fields]) OR "gonadal hormones"[All Fields] OR ("gonadal steroid hormones"[MeSH Terms] OR ("gonadal"[All Fields] AND "steroid"[All Fields] AND "hormones"[All Fields]) OR "gonadal steroid hormones"[All Fields]) OR (("sexual behavior"[MeSH Terms] OR ("sexual"[All Fields] AND "behavior"[All Fields]) OR "sexual behavior"[All Fields] OR "sexual"[All Fields] OR "sexually"[All Fields] OR "sexualities"[All Fields] OR "sexuality"[MeSH Terms] OR "sexuality"[All Fields] OR "sexualization"[All Fields] OR "sexualize"[All Fields] OR "sexualized"[All Fields] OR "sexualizing"[All Fields] OR "sexuals"[All Fields]) AND ("hormon"[All Fields] OR "hormonal"[All Fields] OR "hormonally"[All Fields] OR "hormonals"[All Fields] OR "hormone s"[All Fields] OR "hormones"[Pharmacological Action] OR "hormones"[MeSH Terms] OR "hormones"[All Fields] OR "hormone"[All Fields] OR "hormons"[All Fields]))) AND ("kidney failure, chronic"[MeSH Terms] OR ("kidney"[All Fields] AND "failure"[All Fields] AND "chronic"[All Fields]) OR "chronic kidney failure"[All Fields] OR ("chronic"[All Fields] AND "renal"[All Fields] AND "failure"[All Fields]) OR "chronic renal failure"[All Fields] OR ("blood urea nitrogen"[MeSH Terms] OR ("blood"[All Fields] AND "urea"[All Fields] AND "nitrogen"[All Fields]) OR "blood urea nitrogen"[All Fields]) OR ("haemofiltration"[All Fields] OR "hemofiltration"[MeSH Terms] OR "hemofiltration"[All Fields] OR "hemofiltrated"[All Fields] OR "hemofiltrations"[All Fields]) OR ("kidney failure, chronic"[MeSH Terms] OR ("kidney"[All Fields] AND "failure"[All Fields] AND "chronic"[All Fields]) OR "chronic kidney failure"[All Fields] OR ("chronic"[All Fields] AND "kidney"[All Fields] AND "failure"[All Fields])) OR (("renal"[All Fields] OR "renals"[All Fields]) AND "dialysis*"[All Fields]) OR "uremia*"[All Fields])) |
| Embase | #13. #6 AND #11 AND #12  #12. #7 OR #8 OR #9 OR #10  #11. 'prolactin'/exp  #10. 'progesterone':ti,ab  #9. 'gonadal steroid hormones':ti,ab  #8. 'gonadal hormones':ti,ab  #7. 'sex hormone'/exp  #6. #1 OR #2 OR #3 OR #4 OR #5  #5. 'uremia*':ti,ab  #4. 'renal dialysis*':ti,ab  #3. 'hemofiltration':ti,ab  #2. 'blood urea nitrogen':ti,ab  #1. 'chronic kidney failure'/exp |
| Web of Science | 1: TS=(Chronic renal failure OR Blood Urea Nitrogen OR Hemofiltration OR Chronic Kidney Failure OR Renal Dialysis* OR Uremia*)  2: TS=(Gonadal Hormones OR Gonadal Steroid Hormones OR Sexual Hormones OR Prolactin)  3: #2 AND #1 |
| Cochrane library | #1 ("chronic renal failure") OR (Blood Urea Nitrogen):ti,ab,kw OR (Hemofiltration):ti,ab,kw OR (Chronic Kidney Failure):ti,ab,kw OR (Renal Dialysis*):ti,ab,kw (Word variations have been searched)  #2 (Uremia*):ti,ab,kw (Word variations have been searched)  #3 (Gonadal Hormones) OR (Gonadal Steroid Hormones):ti,ab,kw OR (Sexual Hormones):ti,ab,kw (Word variations have been searched)  #4 #1 or #2  #5 #3 and #4 |

**Supplemental Table 2 (STable2)**. Characters of included studies and patients.

| Study ID | Area and  units | Groups | n | Age  (years) | Drugs might interfering | Time to obtain blood samples ( Menstruating) | Hormones measured | Menopause or amenorrhea;  Ratio or % | Time for HD and/or after RT (months) |
| --- | --- | --- | --- | --- | --- | --- | --- | --- | --- |
| Morley JE 1979 | South Africa  1 unit | HD  Ct  RT | 12  8  5 | 27.6±5.9  Pre-mp  25±7.3 | Met-dopa (HD,RT)  Phenobarb (HD)  Reserpine (RT)  Hygroton (RT); | follicular phase | LH, FSH, E2, P, PRL | HD: 4/12  Ct: 0/8  RT: 1/5 | HD: NA  RT:≥15 |
| Lim VS  1980 | Chicago  1 unit | HD(Re)  HD (Mp)  Ct | 17  7  12 | 18-42  52-59  25-41 | No drugs Interfering | HD: NA  Ct: follicular phase | LH, FSH, E2, P, PRL | HD(Re ):10/17  HD(Mp):7/7  Ct: 0/12 | HD (Re and Mp)  6-48 |
| Zingraff J  1982 | France  1 unit | HD(Re)  HD(Ir) | 6  13 | 39.3±9.0  26.8±7.9 | No hormonal and anti-hypertensive therapy; | During the half of the menstrual cycle | LH, FSH | HD(Re):0/6  HD(Ir):7/13 | HD (Re+Ir)  50±22.25 (2-91) |
| Mantouvalos H 1984 | Greece  1 unit | HD  Ct | 12  50 | 36±9  Pre-mp | No drugs Interfering; | Prior or after dialysis | LH, FSH, E2, P | HD: 5/12  Ct: 0/50 | HD:  13-48 |
| KoutsikosD 1990 | Greece  1 unit | HD (Re)  Ct (Re)  RT (Re) | 2  19  10 | 24,36  32±5  34.7±6.1 | Pn (RT) | follicular phase | LH, FSH, E2, PRL | HD: 0/2  Ct: 0/19  RT: 0/10 | HD:  44.5±28  (5-78)  RT:≥24 |
|  |  | HD (Mp)  Ct (Mp)  RT(Mp) | 11  11  8 | 59.4±4.7  54±5  52.7±5.4 |  |  |  | HD: 11/11  Ct: 11/11  RT: 8/8 |  |
| Akizawa T  1990 | Japan  1 unit | HD  Ct | 10  10 | 40.9 ± 5.3  Age-matched | NA | Before the 1st dialysis after complation of menstruation. | LH, FSH, E2, P, PRL | HD: 0/10  Ct: 0/10 | HD:1-69 |
| Kawashima R 1998 | Japan  1 unit | HD(Re)  HD(Ir) | 13  9 | 38.7±6.7  35±4.3 | No hormonal therapy ≥3 months; | Every other day for 1 month. | LH, FSH, E2, PRL | HD(Re):0/13  HD(Ir): NA | HD(Re+Ir)  53.4±34.3 (7-144) |
| Weisinger JR 2000 | Venezuela  1 unit | HD(Re)  HD(Ir) | 43  31 | 35.4±9.2  38.2±8.9 | No hormonal and corticosteroids therapy recently; | NA | LH,FSH,E2 | HD(Re):0/43  HD(Ir):31/31 | HD(Re): 59.1±46.3  HD(Ir): 53.8±39.1 |
| Park JS  2000 | Korea  1 unit | HD  Ct | 33  32 | 40-73  44-78 | No hormone replacement therapy | NA | E2 | 100% | HD:  ≥6 |
| Matuszkiewicz-Rowinska J 2004 | Poland  6 units | HD  Ct | 75  33 | 35±8  35±6 | No sex hormone or corticosteroids therapy≥5years; | follicular phase  ( FSH, LH, E2),  luteal phase (P) | LH, FSH, E2, P, PRL | HD: 25/75  Ct: 0/33 | HD:  32±30 |
| Song YS  2007 | Korea  1 unit | HD  CT | 38  37 | 46.1±7.4  46.0±10.9 | No hormone therapy≥ 5 years; EPO, NA | The same time of a day | E2, PRL | HD:78.9%  Ct::27% | HD:  96±63 |
| Basok EK  2009 | Turkey  1 unit | HD  Ct  RT | 24  20  20 | 43.1±12.4  32.8± 8.4  36.5± 8.6 | NA | HD: before dialysis  Ct: NA  RT: NA | LH, FSH, E2, PRL | HD: 41.7%  Ct: 5%  RT: 15% | HD: NA  RT:＞6 |
| Arıkan, D. C.  2011 | Turkey  1 unit | HD(Re, Ir)  CT | 30  30 | 34.17± 6.59  32.63± 5.67 | Patients using hormonal drug or oral contraceptives were excluded | follicular phase | FSH, LH, E2, PRL | HD: 43.3%  CT: NA | HD: 18-264 |
| Kurdoglu Z 2012 | Turkey  1 unit | HD  Ct | 25  30 | 45.0±10.6  42.3±10.8 | NA | The same time of a day, before dialysis | LH, FSH, E2, PRL | HD: NA  Ct: NA | HD:＞6 |
| Kim JH  2014 | Korea  1 unit | HD  Ct | 18  36 | 47.9±6.8  48.2± 6.9 | No hormonal therapy; | The same time of a day, before dialysis | E2 | HD: 13/18  Ct: 10/36 | HD:  88.8±83.9 |
| Altunoglu A. 2014 | Turkey  1 unit | HD  RT  Ct | 46  47  36 | 27-63  18-55  23-53 | No patient on oral contraceptive and sirolimus treatment | measured following 8 hour fast | LH, FSH, E2, PRL | NA | HD:NA  RT:6 |
| Esen B  2015 | Turkey  1 unit | HD  Ct | 26  20 | 49.1±9.87  48.3±7.22 | NA | follicular phase | LH, FSH, E2, PRL | HD:33%  Ct: 30% | NA |
| Grossmann M 2015 | Australia  1 unit | HD  RT | 41  26 | 52-73  50-62 | NA | NA | E2 | NA | HD: 1.8-9.0  RT: 1.7-13.4 |
| Lin CT  2016 | China  1 unit | HD (Re)  RT (Re) | 11  18 | 34.6±5.4  34.5±4.8 | No hormonal therapy≥ 2 years | follicular phase  ( FSH, LH, PRL),  luteal phase (P) | LH, FSH, P, PRL | HD: 0/11  RT: 0/18 | HD: 52.2±7.8  RT:≥6 |
| Stoumpos S  2018 | The UK.  1 unit | HD  RT | 26  25 | 34± 4.9  31.2± 5.8 | Hormonal contraception | A random day of menstrual circle | LH, FSH, E2, P, PRL | HD: 26.9%  RT: 12% | NA |
| Fayed A  2019 | Egypt  1 unit | HD  Ct | 60  50 | 30± 3.9  29.6±3 | No hormonal and oral contraception therapy | follicular phase | LH, FSH, PRL | HD: 45%(Ir)  Ct: 0/50 | HD: 26.9±8.75 |
| Fathalla, M.  2021 | Sudan  1 unit | HD  CT | 20  50 | 18-55  32.2±10.0 | patients using PRL drugs and those taking anabolizing androgen were excluded. | between 8 and 10 am after an overnight fast | FSH, LH, PRL | NA | HD:4-14 |

**Abbreviations:** n, number; y, years; Re, women with regular menses; Mp, women after menopausal; Ir, women with irregular menses. HD, hemodialysis; Ct, healthy control; RT, renal transplantation; NA, not applicable; Pn, Prednisone or medicine of this kind; Met-dopa, methyldopa or medicine of this kind.

**Supplemental Table 3 (STable3)**. Criteria adopted for Risk of Bias assessment.

| **Domains** | **Criteria** |
| --- | --- |
| Bias due to confounding | Factors considered mandatory in order to judge a study at moderate risk of bias are: **age, time to obtain blood samples, drugs used due to kidney disease or complications might interfering hormones levels.**  Factors considered mandatory to judge a study at low risk of bias are: **in regular hemodialysis or not and duration after renal transplantation.**  To be considered at low risk of bias, a study must include **age, time to obtain blood samples, drugs used due to kidney disease or complications might interfering hormones level, reporting in regular hemodialysis, and reporting the duration after renal transplantation.** |
| Bias in selecting participants in the study | Studies included patients firstly and then, tested hormones. If the main confounding factors in the first domain were already considered, selection of subjects was unrelated to either exposure or outcome. So, there should be no concern about the selecting bias. |
| Bias in exposure classification | There should be no concern about misclassification bias because they are all clinically diagnosed chronic renal failure needing hemodialysis or they already completed renal transplantation. |
| Bias in departure from intended exposure | There should be no concern about departure from intended exposure because the disease is not reversible and that a regular hemodialysis and duration after renal transplantation have been considered at the first domain of confounding bias. |
| Bias due to missing data | Studies with less than 10% are considered at low risk bias of missing data. |
| Bias in outcome measurement | Different methods testing hormones could contribute to the bias in outcome measurement but it is considered at low risk. |
| Bias in selection of reported results | Evidence that results have not been selected. Clear reporting of statistical methods. |
| Overall risk of bias | If at least one domain was found at risk of bias, the overall risk was considered high. If more than one domain was found at moderate risk of bias, the overall risk was considered moderate. If all domains were at low risk of bias, the overall risk was considered low. |

**Supplemental Table 4 (STable4)**. Detailed of Risk of Bias (RoB) assessment for all included studies.

| **Bias item** | **Risk of bias** | **Rationale** |  |
| --- | --- | --- | --- |
| **(** **Morley JE et al. 1979)** | | | |
| Bias due to confounding | Moderate | Baseline characteristics of patients were provided in this study and all participants were aged 37 or less, thus they were all pre-menopausal women. Time to obtain blood samples were reported as follows: All the tests were carried out between dialyses. In those patients who were menstruating the test was performed during the first 10 days of the cycle. Drugs might interfering hormones levels such as prednisone, reserpine and methyldopa were also reported, but they were not adjusted between groups. Patients included were undergoing regular hemodialysis and the duration after renal transplantation were all longer than 6 months. |  |
| Bias in selection of participants into the study | Low | The study included patients firstly and then, tested hormones. If the main confounding factors in the first domain were already considered, selection of subjects was unrelated to either exposure or outcome. So, there should be no concern about the selecting bias. |  |
| Bias in classification of exposures | Low | Exposure (chronic renal failure undergoing regular hemodialysis or after renal transplantation with stable renal function) was confirmed prior to the outcome (hormones measurement); thus, it was not possible for classification of exposure to have been affected by the knowledge of the outcome. |  |
| Bias due to departures from intended exposures | Low | The exposure was completed at recruitment, while the outcome was assessed after recruitment. A regular hemodialysis and the duration after renal transplantation have been considered and it’s impossible for patients to departures from intended exposure. |  |
| Bias due to missing data | Low | All included patients had measured hormones. |  |
| Bias in measurement of outcomes | Low | No risk of bias. Blood was taken by researchers and hormones were tested by machine. Different methods testing hormones among studies could contribute to the bias in outcome measurement but it is considered at low risk. |  |
| Bias in selection of reported results | Low | Study results such as hormones values of all included patients were fully reported, and also some confounding information. Statistical methods reported in the methods section were used and presented in the results. |  |
| Overall bias | **Moderate** | Possible bias in confounding. |  |
| **(** **Akizawa T et al. 1990)** | | | |
| Bias due to confounding | Low | In this study, all included women had a biphasic basal body temperature and regular menstruation despite their age ranged between 33 to 48 years. Basal characteristics of the participants are reported in Table 1. The blood samples were taken before the 1st dialysis after the completion of menstruation, the same as in the follicular phase. This study aimed at improving endocrinological functions by administrating recombinant human erythropoietin (r-HuEPO) in ameliorating renal anemia. We only choose the values of hormones before administrating r-HuEPO. This study did not mention if there existed drugs interfering hormones levels, but considering that they all had a biphasic basal body temperature and regular menstruation, the possibility was small. All patients in this study were in regular hemodialysis. |  |
| Bias in selection of participants into the study | Low | The study included patients firstly and tested hormones secondly. If the main confounding factors in the first domain were already considered, selection of subjects was unrelated to either exposure or outcome. So, there should be no concern about the selecting bias. |  |
| Bias in classification of exposures | Low | Exposure (chronic renal failure undergoing regular hemodialysis) was confirmed prior to the outcome (hormones measurement); and it was not possible for classification of exposure to have been affected by the knowledge of the outcome. |  |
| Bias due to departures from intended exposures | Low | The exposure (patient undergoing regular hemodialysis) was impossible to departure before hormones taken in this study. |  |
| Bias due to missing data | Low | This study only included 10 patients and none of their hormones values was missed (shown in table 2). |  |
| Bias in measurement of outcomes | Low | No risk of bias. Blood was taken by researchers and hormones were tested by machine. Different methods testing hormones among studies could contribute to the bias in outcome measurement but it is considered at low risk. |  |
| Bias in selection of reported results | Low | Study results fully reported. Statistical methods reported in the methods section were used and presented in the results. |  |
| Overall bias | **Low** | No major concerns for risk of bias assessment. |  |
| **(** **Koutsikos D et al. 1990)** | | | |
| Bias due to confounding | Low | Results were stratified by menstruation status. The age was reported as mean ± standard deviation. Hormones of menstruating women were tested during follicular phase. Propranolol, prazosin, or amethyldopa were stopped 2 months before the study and replaced by calcium channel blockers or ACE inhibitors. No patient suffered active or chronic liver disease, malignancies, SLE, or diabetes mellitus. None was taking any kind of hormonal therapy. All patients were undergoing regular hemodialysis or their graft function had been stabilized for at least 24 months. |  |
| Bias in selection of participants into the study | Low | The study included patients firstly and tested hormones secondly. If the main confounding factors in the first domain were already considered, selection of subjects was unrelated to either exposure or outcome. So, there should be no concern about the selecting bias. |  |
| Bias in classification of exposures | Low | Exposure (chronic renal failure undergoing regular hemodialysis or after renal transplantation with stable renal function) was confirmed prior to the outcome (hormones measurement); thus, it was not possible for classification of exposure to have been affected by the knowledge of the outcome. |  |
| Bias due to departures from intended exposures | Low | The exposure (patient with stable graft renal function or undergoing regular hemodialysis) was impossible to departure before hormones taken in this study. |  |
| Bias due to missing data | Low | Hormones values of all included patients were obtained (shown in table 1). |  |
| Bias in measurement of outcomes | Low | No risk of bias. Blood was taken by researchers and hormones were tested by machine. Different methods testing hormones among studies could contribute to the bias in outcome measurement but it is considered at low risk. |  |
| Bias in selection of reported results | Low | Study results fully reported (Table 1). |  |
| Overall bias | **Low** | No major concerns for risk of bias assessment. |  |
| **(** **Mantouvalos H et al. 1984)** | | | |
| Bias due to confounding | Moderate | The included patients aged from 18 to 45 years with a mean of 36 year. Blood was taken from each patient by venipuncture, once a week for 8 weeks, prior to and just after dialysis. The blood samples were not taken during follicular phase and this contributed to confounding bias. All included patients had not been given any drugs which could have interfered with measurements for at least the previous 12 months. All included patients undergoing regular hemodialysis. |  |
| Bias in selection of participants into the study | Low | The study included patients firstly and tested hormones secondly. If the main confounding factors in the first domain were already considered, selection of subjects was unrelated to either exposure or outcome. So, there should be no concern about the selecting bias. |  |
| Bias in classification of exposures | Low | Exposure was assessed prior to the outcome; and it was not possible for classification of exposure (in regular hemodialysis or not; after renal transplantation with stable renal function or not) to have been affected by the knowledge of the outcome (hormones values). |  |
| Bias due to departures from intended exposures | Low | The exposure (patient undergoing regular hemodialysis) was impossible to departure before hormones taken in this study. |  |
| Bias due to missing data | Low | Hormones values of all included patients were obtained (shown in table II). |  |
| Bias in measurement of outcomes | Low | No risk of bias. Blood was taken by researchers and hormones were tested by machine. Different methods testing hormones among studies could contribute to the bias in outcome measurement but it is considered at low risk. |  |
| Bias in selection of reported results | Low | Study results fully reported (Table II). Statistical methods reported in the methods section were used and presented in the results. |  |
| Overall bias | **Moderate** | Possible bias due to confounding (time to obtain blood samples). |  |
| **(Zingraff J et al. 1982)** | | | |
| Bias due to confounding | Low | The age of every patients included in this study was given. Time to obtain every blood sample was recorded in this study. None of this patients received hormonal or anti-hypertensive therapy which eliminating the interfering of drugs. All patients undergoing regular hemodialysis. There was no renal transplantation patients included. |  |
| Bias in selection of participants into the study | Low | The study included patients firstly and tested hormones secondly. If the main confounding factors in the first domain were already considered, selection of subjects was unrelated to either exposure or outcome. So, there should be no concern about the selecting bias. |  |
| Bias in classification of exposures | Low | Exposure (chronic renal failure undergoing regular hemodialysis) was confirmed prior to the outcome (hormones measurement); thus, it was not possible for classification of exposure to have been affected by the knowledge of the outcome. |  |
| Bias due to departures from intended exposures | Low | The exposure (patient undergoing regular hemodialysis) was impossible to departure before hormones taken in this study. |  |
| Bias due to missing data | Low | Hormones values of all included patients were obtained. |  |
| Bias in measurement of outcomes | Low | No risk of bias. Blood was taken by researchers and hormones were tested by machine. Different methods testing hormones among studies could contribute to the bias in outcome measurement but it is considered at low risk. |  |
| Bias in selection of reported results | Low | Study results fully reported (Table I to IV). |  |
| Overall bias | **Low** | No major concerns for risk of bias assessment. |  |
| **(** **Lim VS et al. 1980)** | | | |
| Bias due to confounding | Moderate | This study included both premenopausal and postmenopausal women. The premenopausal women ranged from 18 to 42 years old and the postmenopausal women from 52 to 59 years. None of the patients received androgens, estrogen, or medications reported to alter the hypothalamic-pituitary regulation of gonadotropin or prolactin secretion (for example, methyldopa, reserpine, clonidine, or phenothiazines). Time to obtain blood samples was not mentioned. All patients received regular hemodialysis. |  |
| Bias in selection of participants into the study | Low | The study included patients firstly and tested hormones secondly. If the main confounding factors in the first domain were already considered, selection of subjects was unrelated to either exposure or outcome. So, there should be no concern about the selecting bias. |  |
| Bias in classification of exposures | Low | Exposure was assessed prior to the outcome and it was not possible for classification of exposure to have been affected by the knowledge of the outcome. |  |
| Bias due to departures from intended exposures | Low | The exposure (patient undergoing regular hemodialysis) was impossible to departure before hormones taken in this study. |  |
| Bias due to missing data | Low | Hormones values of all included patients were obtained. |  |
| Bias in measurement of outcomes | Low | No risk of bias. Blood was taken by researchers and hormones were tested by machine. Different methods testing hormones among studies could contribute to the bias in outcome measurement but it is considered at low risk. |  |
| Bias in selection of reported results | Low | Study results fully reported. |  |
| Overall bias | **Moderate** | There might exist the confounding bias. |  |
| **(** **Kawashima R et al. 1998)** | | | |
| Bias due to confounding | Moderate | The age of included patients ranged 24 to 48 years. Hormones were determined every other day for 1 month, not mentioning the exact menstrual circle phase. Before blood sampling, the author confirmed that the patient had not received any hormone preparation during the previous 3 months. All patients were undergoing regular hemodialysis. |  |
| Bias in selection of participants into the study | Low | The study included patients firstly and tested hormones secondly. If the main confounding factors in the first domain were already considered, selection of subjects was unrelated to either exposure or outcome. So, there should be no concern about the selecting bias. |  |
| Bias in classification of exposures | Low | Exposure was assessed prior to the outcome and it was not possible for classification of exposure to have been affected by the knowledge of the outcome. |  |
| Bias due to departures from intended exposures | Low | The exposure (patient undergoing regular hemodialysis) was impossible to departure before hormones taken in this study. |  |
| Bias due to missing data | Low | Hormones values of all included patients were obtained (Table 1). |  |
| Bias in measurement of outcomes | Low | No risk of bias. Blood was taken by researchers and hormones were tested by machine. Different methods testing hormones among studies could contribute to the bias in outcome measurement but it is considered at low risk. |  |
| Bias in selection of reported results | Low | Study results fully reported. |  |
| Overall bias | **Moderate** | Possible confounding bias existed. |  |
| **(Weisinger JR et al. 2000)** | | | |
| Bias due to confounding | Moderate | The age of included patients was shown in table 1. The time to obtain hormones was not mentioned. Patients who recently had been treated with estrogen, progesterone, corticosteroids, anticonvulsants, fluoride, bisphospho-follicle stimulating hormone; parathynates, or calcitonin were excluded from the study which excluded the drugs interfering. All patients included were undergoing regular hemodialysis. |  |
| Bias in selection of participants into the study | Low | The study included patients firstly and tested hormones secondly. If the main confounding factors in the first domain were already considered, selection of subjects was unrelated to either exposure or outcome. So, there should be no concern about the selecting bias. |  |
| Bias in classification of exposures | Low | Exposure was assessed prior to the outcome and it was not possible for classification of exposure to have been affected by the knowledge of the outcome. |  |
| Bias due to departures from intended exposures | Low | The exposure (patient undergoing regular hemodialysis) was impossible to departure before hormones taken in this study. |  |
| Bias due to missing data | Low | This study excluded postmenopausal women and the remaining women were included in analysis. The data of all included patients were shown in table 1 and table 2. |  |
| Bias in measurement of outcomes | Low | No risk of bias. Blood was taken by researchers and hormones were tested by machine. Different methods testing hormones among studies could contribute to the bias in outcome measurement but it is considered at low risk. |  |
| Bias in selection of reported results | Low | Study results fully reported, baseline characteristics (Table 1), hormones values (Table 2). |  |
| Overall bias | **Moderate** | Possible bias in not mentioning time to obtain blood samples. |  |
| **(Park JS et al. 2000)** | | | |
| Bias due to confounding | Low | The age of included patients was shown in table 1. The time to obtain hormones was not mentioned, but all patients didn’t have mensturation for at least 1 year. Patients who had history of previous hormone replacement therapy were excluded. All patients included were undergoing regular hemodialysis at least 6 months. |  |
| Bias in selection of participants into the study | Moderate | This study selected patients and control with oestradiol less than 25pg/ml. Participators were comparable inner study but there might existed bias among studies. |  |
| Bias in classification of exposures | Low | It was not possible for classification of exposure to have been affected by the knowledge of the outcome. |  |
| Bias due to departures from intended exposures | Low | The exposure (patient undergoing regular hemodialysis) was impossible to departure before hormones taken in this study. |  |
| Bias due to missing data | Low | 65 of 70 patients completed the study which was acceptable and didn’t causing the bias. |  |
| Bias in measurement of outcomes | Low | No risk of bias. Blood was taken by researchers and hormones were tested by machine. Different methods testing hormones among studies could contribute to the bias in outcome measurement but it is considered at low risk. |  |
| Bias in selection of reported results | Low | Study results fully reported. |  |
| Overall bias | **Moderate** | Possible selecting bias among studies. |  |
| **(Matuszkiewicz-Rowinska J et al. 2004)** | | |  |
| Bias due to confounding | **Low** | The age of all included patients was shown in table 3. In this study, the prolactin, FSH, LH, and E2 were tested during follicular phase and the progesterone was tested during luteal phase. No one had been treated with sex hormones or corticosteroids for at least 5 years. All included patients were undergoing regular hemodialysis. |  |
| Bias in selection of participants into the study | **Low** | The study included patients firstly and tested hormones secondly. If the main confounding factors in the first domain were already considered, selection of subjects was unrelated to either exposure or outcome. So, there should be no concern about the selecting bias. |  |
| Bias in classification of exposures | **Low** | Exposure was assessed prior to the outcome and it was not possible for classification of exposure to have been affected by the knowledge of the outcome. |  |
| Bias due to departures from intended exposures | **Low** | The exposure (patient undergoing regular hemodialysis) was impossible to departure before hormones taken in this study. |  |
| Bias in measurement of outcomes | **Low** | No risk of bias. Blood was taken by researchers and hormones were tested by machine. Different methods testing hormones among studies could contribute to the bias in outcome measurement but it is considered at low risk. |  |
| Bias in selection of reported results | **Low** | Study results seem fully reported (table 1, table 2 and table 3). |  |
| Overall bias | **Low** | No major concerns for risk of bias assessment. |  |
| **(****Song YS et al. 2008)** | | |  |
| Bias due to confounding | **Moderate** | The age and hormones values of hemodialysis and control women were shown in table 1. Blood samples were always drawn at the same time of the day not mentioning the exact phase in menstrual circle. No one had been treated with sex hormones for at least 5 years. Patients in this study all undergoing regular hemodialysis. |  |
| Bias in selection of participants into the study | **Low** | This study included 38 consecutive married women (aged 30 to 64 years) with chronic renal failure, who were receiving hemodialysis, and were eligible and willing to participate in the study. |  |
| Bias in classification of exposures | **Low** | Exposure was assessed prior to the outcome and it was not possible for classification of exposure to have been affected by the knowledge of the outcome. |  |
| Bias due to departures from intended exposures | **Low** | The exposure (patient undergoing regular hemodialysis) was impossible to departure before hormones taken in this study. |  |
| Bias in measurement of outcomes | **Low** | No risk of bias. Blood was taken by researchers and hormones were tested by machine. Different methods testing hormones among studies could contribute to the bias in outcome measurement but it is considered at low risk. |  |
| Bias in selection of reported results | **Low** | Study results seem fully reported (table 1) |  |
| Overall bias | **Moderate** | Possible bias in confounding. |  |
| **(Basok EK et al. 2009)** | | |  |
| Bias due to confounding | **Moderate** | The age and other baseline characteristics were shown in table 1. Blood samples were taken before dialysis not mentioning the exact phase in menstrual circle. Not mentioning if there were medicines interfering the hormones values. Patients needing hemodialysis were in a regular basis and patients in transplanted group had performed renal transplantation at least 6 months. |  |
| Bias in selection of participants into the study | **Low** | This study included 106 women, including 24 in hemodialysis and 20 after renal transplantation. The study included patients firstly and tested hormones secondly. If the main confounding factors in the first domain were already considered, selection of subjects was unrelated to either exposure or outcome. So, there should be no concern about the selecting bias. |  |
| Bias in classification of exposures | **Low** | Exposure was assessed prior to the outcome and it was not possible for classification of exposure to have been affected by the knowledge of the outcome. |  |
| Bias due to departures from intended exposures | **Low** | The exposure (chronic renal failure patients undergoing regular hemodialysis or after renal transplantation) was impossible to departure before hormones taken in this study. |  |
| Bias in measurement of outcomes | **Low** | No risk of bias. Blood was taken by researchers and hormones were tested by machine. Different methods testing hormones among studies could contribute to the bias in outcome measurement but it is considered at low risk. |  |
| Bias in selection of reported results | **Low** | Study results seem fully reported (table 1, table 2 et.al.) |  |
| Overall bias | **Moderate** | Possible bias in confounding. |  |
| **(Arıkan, D. C. et al. 2011)** | | |  |
| Bias due to confounding | **Low** | The age and other baseline characteristics were shown in table 1 and table 2. Venous blood was obtained from the hemodialysis patients on the 5th day of menstruation (if they would have dialysis on the 5th day of menstrual cycle just before the dialysis). Patients using hormonal drug or oral contraceptives for contraception were excluded from the study. Patients needing hemodialysis were in a regular basis. |  |
| Bias in selection of participants into the study | **Low** | This study included 60 women, including 30 in hemodialysis and 30 after renal transplantation. The study included patients firstly and tested hormones secondly. If the main confounding factors in the first domain were already considered, selection of subjects was unrelated to either exposure or outcome. So, there should be no concern about the selecting bias. |  |
| Bias in classification of exposures | **Low** | Exposure was assessed prior to the outcome and it was not possible for classification of exposure to have been affected by the knowledge of the outcome. |  |
| Bias due to departures from intended exposures | **Low** | The exposure (chronic renal failure patients undergoing regular hemodialysis) was impossible to departure before hormones taken in this study. |  |
| Bias in measurement of outcomes | **Low** | No risk of bias. Blood was taken by researchers and hormones were tested by machine. Different methods testing hormones among studies could contribute to the bias in outcome measurement but it is considered at low risk. |  |
| Bias in selection of reported results | **Low** | Study results seem fully reported (table 1, table 2 et.al.) |  |
| Overall bias | **Low** | No major concerns for risk of bias assessment. |  |
| **(Kurdoglu Z et al. 2012)** | | |  |
| Bias due to confounding | **Moderate** | The age of included patients were shown in table 1. The hormones of included patients were taken from each woman between 8 AM and 10 AM (for hemodialysis patients, before the dialysis), not mentioning the exact menstrual circle phase. This study also not mentioned if there existed drugs interfering the hormones values. All patients needing hemodialysis were in a regular routine. |  |
| Bias in selection of participants into the study | **Low** | This study enrolled patients according to inclusion criteria and exclusion criteria and considering. And the study included patients firstly and tested hormones secondly. If the main confounding factors in the first domain were already considered, selection of subjects was unrelated to either exposure or outcome. So, there should be no concern about the selecting bias. |  |
| Bias in classification of exposures | **Low** | Exposure was assessed prior to the outcome and it was not possible for classification of exposure to have been affected by the knowledge of the outcome. |  |
| Bias due to departures from intended exposures | **Low** | The exposure (chronic renal failure patients undergoing regular hemodialysis) was impossible to departure before hormones taken in this study. |  |
| Bias in measurement of outcomes | **Low** | No risk of bias. Blood was taken by researchers and hormones were tested by machine. Different methods testing hormones among studies could contribute to the bias in outcome measurement but it is considered at low risk. |  |
| Bias in selection of reported results | **Low** | Study results seem fully reported. |  |
| Overall bias | **Moderate** | Possible confounding bias existed. |  |
| **(Kim JH et al. 2014)** | | |  |
| Bias due to confounding | **Moderate** | The ages of included patients were shown in table 1. Blood samples were always drawn at the same time of day (08:00 to 10:00 hours), not mentioning the exact menstrual circle phase. Patients were excluded when they had used hormonal replacement therapy within the past 5 years. All patients included undergoing regular hemodialysis. |  |
| Bias in selection of participants into the study | **Low** | This study included a total of 37 consecutive married women with CRF from March 2008 to February 2011, who were on hemodialysis and willing to participate in the study. |  |
| Bias in classification of exposures | **Low** | Exposure was assessed prior to the outcome and it was not possible for classification of exposure to have been affected by the knowledge of the outcome. |  |
| Bias due to departures from intended exposures | **Low** | The exposure (chronic renal failure patients undergoing regular hemodialysis) was impossible to departure before hormones taken in this study. |  |
| Bias in measurement of outcomes | **Low** | No risk of bias. Blood was taken by researchers and hormones were tested by machine. Different methods testing hormones among studies could contribute to the bias in outcome measurement but it is considered at low risk. |  |
| Bias in selection of reported results | **Low** | Study results seem fully reported. |  |
| Overall bias | **Moderate** | Possible confounding bias existed. |  |
| **(Altunoglu A et al. 2014)** | | |  |
| Bias due to confounding | **Moderate** | The ages of included patients were shown in table 1. Blood samples were measured following 8 hour fast, not mentioning the exact menstrual circle phase. Patients were excluded when they were on oral contraceptive and sirolimus treatment and after renal transplantation for < 6 months. All patients included undergoing regular hemodialysis. |  |
| Bias in selection of participants into the study | **Low** | The study included patients firstly and tested hormones secondly. If the main confounding factors in the first domain were already considered, selection of subjects was unrelated to either exposure or outcome. So, there should be no concern about the selecting bias. |  |
| Bias in classification of exposures | **Low** | Exposure was assessed prior to the outcome and it was not possible for classification of exposure to have been affected by the knowledge of the outcome. |  |
| Bias due to departures from intended exposures | **Low** | The exposure (chronic renal failure patients undergoing regular hemodialysis and patients after renal transplantation≥6 month) was impossible to departure before hormones taken in this study. |  |
| Bias in measurement of outcomes | **Low** | No risk of bias. Blood was taken by researchers and hormones were tested by machine. And hormones were tested with the use of standard methods in the routine clinical and hormone laboratory. |  |
| Bias in selection of reported results | **Low** | Study results seem fully reported. |  |
| Overall bias | **Moderate** | Possible confounding bias existed. |  |
| **(****Esen B et al. 2015)** | | |  |
| Bias due to confounding | **Moderate** | The age of included patients was shown in table 4. Fasting blood samples were collected in early follicular phase of female patients and controls. Whether there existed drugs interfering the accuracy of hormones values was not mentioned in this study. If patients were in regular hemodialysis was also not mentioned. |  |
| Bias in selection of participants into the study | **Low** | This study included a total of 53 consecutive patients. |  |
| Bias in classification of exposures | **Low** | Exposure was assessed prior to the outcome and it was not possible for classification of exposure to have been affected by the knowledge of the outcome. |  |
| Bias due to departures from intended exposures | **Low** | The exposure (chronic renal failure patients undergoing regular hemodialysis) was impossible to departure before hormones taken in this study. |  |
| Bias in measurement of outcomes | **Low** | No risk of bias. Blood was taken by researchers and hormones were tested by machine. Different methods testing hormones among studies could contribute to the bias in outcome measurement but it is considered at low risk. |  |
| Bias in selection of reported results | **Low** | Study results seem fully reported. |  |
| Overall bias | **Moderate** | Possible confounding bias existed. |  |
| **(Grossmann M et al. 2015)** | | |  |
| Bias due to confounding | **Moderate** | The age of included patients was shown in table 2. Blood samples were collected at baseline not mentioning the exact time during menstrual circle. Whether there existed drugs interfering the accuracy of hormones values was not mentioned in this study. All patients were in regular hemodialysis and received dialysis for a median of 3.6 year (1.8–9.0), respectively, and in renal transplantation group, they had their transplant surgery 4.9 years (1.7–13.4), respectively. |  |
| Bias in selection of participants into the study | **Low** | If the main confounding factors in the first domain were already considered, selection of subjects was unrelated to either exposure or outcome in this study. |  |
| Bias in classification of exposures | **Low** | Exposure was assessed prior to the outcome and it was not possible for classification of exposure to have been affected by the knowledge of the outcome. |  |
| Bias due to departures from intended exposures | **Low** | The exposure (chronic renal failure patients undergoing regular hemodialysis and after renal transplantation) was impossible to departure before hormones taken in this study. |  |
| Bias in measurement of outcomes | **Low** | No risk of bias. Blood was taken by researchers and hormones were tested by machine. Different methods testing hormones among studies could contribute to the bias in outcome measurement but it is considered at low risk. |  |
| Bias in selection of reported results | **low** | Study results seem fully reported. |  |
| Overall bias | **Moderate** | Possible confounding bias existed. |  |
| **(****Lin CT et al. 2016)** | | |  |
| Bias due to confounding | **Low** | The age of included patients was shown in table 1. Blood samples for the detection of PRL, FSH and LH were collected in the morning on days 3–5 of the cycle (the follicular phase). Blood samples for the detection of progesterone and testosterone were collected 4–6 days before anticipated menses (the luteal phase). This study excluded patients using sex hormones in the prior 2 years. All hemodialysis patients were in a regular dialysis and all patients had a duration after renal transplantation for at least 6 months. |  |
| Bias in selection of participants into the study | **Low** | A total of 184 patients were initially enrolled in this study and Nineteen patients did not complete the final analysis because of the following reasons: not providing the characteristics of the menstrual cycle (6 patients), switching to another renal replacement therapy (8 patients) or missing information about the serum hormonal status (5 patients). If the main confounding factors in the first domain were already considered, selection of subjects was unrelated to either exposure or outcome. So, there should be no concern about the selecting bias. |  |
| Bias in classification of exposures | **Low** | Exposure was assessed prior to the outcome and it was not possible for classification of exposure to have been affected by the knowledge of the outcome. |  |
| Bias due to departures from intended exposures | **Low** | The exposure (chronic renal failure patients undergoing regular hemodialysis) was impossible to departure before hormones taken in this study. |  |
| Bias in measurement of outcomes | **Low** | No risk of bias. Blood was taken by researchers and hormones were tested by machine. Different methods testing hormones among studies could contribute to the bias in outcome measurement but it is considered at low risk. |  |
| Bias in selection of reported results | **Low** | Study results seem fully reported. |  |
| Overall bias | **Low** | No major concerns for risk of bias assessment. |  |
| **(Stoumpos S. et al. 2018)** | | |  |
| Bias due to confounding | **Moderate** | The age of included patients was shown in table 1. The blood samples were collected on a random day during the menstrual cycle and then anonymized and centrifuged. There was similar contraception and steroids using between hemodialysis women and women after renal transplantation. All hemodialysis patients were in a regular dialysis but it was unclear the duration of patients after renal transplantation. |  |
| Bias in selection of participants into the study | **Low** | If the main confounding factors in the first domain were already considered, selection of subjects was unrelated to either exposure or outcome. So, there should be no concern about the selecting bias. |  |
| Bias in classification of exposures | **Low** | Exposure was assessed prior to the outcome and it was not possible for classification of exposure to have been affected by the knowledge of the outcome. |  |
| Bias due to departures from intended exposures | **Low** | The exposure (chronic renal failure patients undergoing regular hemodialysis) was impossible to departure before hormones taken in this study. |  |
| Bias in measurement of outcomes | **Low** | No risk of bias. Blood was taken by researchers and hormones were tested by machine. Different methods testing hormones among studies could contribute to the bias in outcome measurement but it is considered at low risk. |  |
| Bias in selection of reported results | **Low** | Study results seem fully reported. |  |
| Overall bias | **Moderate** | Possible confounding bias existed. |  |
| **(Fayed A et al. 2019)** | | |  |
| Bias due to confounding | **Low** | The age of included patients was shown in table 1. FSH and LH were done on the third day of cycle (follicular phase) if the patient had no amenorrhea. Patients using hormonal replacement therapy or oral contraception were excluded. All patients undergone regular hemodialysis. |  |
| Bias in selection of participants into the study | **Low** | If the main confounding factors in the first domain were already considered, selection of subjects was unrelated to either exposure or outcome. So, there should be no concern about the selecting bias. |  |
| Bias in classification of exposures | **Low** | Exposure was assessed prior to the outcome and it was not possible for classification of exposure to have been affected by the knowledge of the outcome. |  |
| Bias due to departures from intended exposures | **Low** | The exposure (chronic renal failure patients undergoing regular hemodialysis) was impossible to departure before hormones taken in this study. |  |
| Bias in measurement of outcomes | **Low** | No risk of bias. Blood was taken by researchers and hormones were tested by machine. Different methods testing hormones among studies could contribute to the bias in outcome measurement but it is considered at low risk. |  |
| Bias in selection of reported results | **Low** | Study results seem fully reported. |  |
| Overall bias | **Low** | No major concerns for risk of bias assessment. |  |
| **(Fathalla, M. et al. 2021)** | | |  |
| Bias due to confounding | **Moderate** | The age of included patients was shown in table 1. Blood samples were the serum collected through centrifugation that was drawn from all subjects between 8 and 10 am after an overnight fast. Patients using PRL drugs and those taking anabolizing androgen were excluded. All hemodialysis patients were in a regular dialysis. |  |
| Bias in selection of participants into the study | **Low** | If the main confounding factors in the first domain were already considered, selection of subjects was unrelated to either exposure or outcome. So, there should be no concern about the selecting bias. |  |
| Bias in classification of exposures | **Low** | Exposure was assessed prior to the outcome and it was not possible for classification of exposure to have been affected by the knowledge of the outcome. |  |
| Bias due to departures from intended exposures | **Low** | The exposure (chronic renal failure patients undergoing regular hemodialysis) was impossible to departure before hormones taken in this study. |  |
| Bias in measurement of outcomes | **Low** | No risk of bias. Blood was taken by researchers and hormones were tested by machine. Different methods testing hormones among studies could contribute to the bias in outcome measurement but it is considered at low risk. |  |
| Bias in selection of reported results | **Low** | Study results seem fully reported. |  |
| Overall bias | **Moderate** | Possible confounding bias existed. |  |

**Supplemental Table 5 (STable5)**. Summary Risk of Bias (RoB) assessment with overall study-level risk of bias.

| **Studies** | **Design** | Bias due to confounding | Bias in selecting participants in the study | Bias in exposure classification | Bias in departure from intended exposure | Bias due to missing data | Bias in outcome measurement | Bias in selection of reported results | **Study-level RoB Judgment** |
| --- | --- | --- | --- | --- | --- | --- | --- | --- | --- |
| ( Morley JE et al. 1979) | Cross-sectional | Moderate | Low | Low | Low | Low | Low | Low | **Moderate** |
| ( Akizawa T et al. 1990) | Cross-sectional | Low | Low | Low | Low | Low | Low | Low | **low** |
| ( Koutsikos D et al. 1990) | Cross-sectional | Low | Low | Low | Low | Low | Low | Low | **Low** |
| ( Mantouvalos H et al. 1984) | Cross-sectional | Moderate | Low | Low | Low | Low | Low | Low | **Moderate** |
| ( Zingraff J et al. 1982) | Cross-sectional | Low | Low | Low | Low | Low | Low | Low | **Low** |
| ( Lim VS et al. 1980) | Cross-sectional | Moderate | Low | Low | Low | Low | Low | Low | **Moderate** |
| ( Kawashima R et al. 1998) | Cross-sectional | Moderate | Low | Low | Low | Low | Low | Low | **Moderate** |
| (Weisinger JR et al. 2000) | Cross-sectional | Moderate | Low | Low | Low | Low | Low | Low | **Moderate** |
| (Park JS et al. 2000) | Cross-sectional | Low | Moderate | Low | Low | Low | Low | Low | **Moderate** |
| (Matuszkiewicz-Rowinska J et al. 2004) | Cross-sectional | Low | Low | Low | Low | Low | Low | Low | **Low** |
| (Song YS et al. 2008) | Cross-sectional | Moderate | Low | Low | Low | Low | Low | Low | **Moderate** |
| (Basok EK et al. 2009) | Cohort | Moderate | Low | Low | Low | Low | Low | Low | **Moderate** |
| (Filocamo MT et al. 2009) | Cross-sectional | Low | Low | Low | Low | Low | Low | Low | **Low** |
| (Arıkan, D. C. et al. 2011) | Cross-sectional | low | Low | Low | Low | Low | Low | Low | **Low** |
| (Kurdoglu Z et al. 2012) | Cross-sectional | Moderate | Low | Low | Low | Low | Low | Low | **Moderate** |
| (Kim JH et al. 2014) | Cross-sectional | Moderate | Low | Low | Low | Low | Low | Low | **Moderate** |
| (Esen B et al. 2015) | Cross-sectional | Moderate | Low | Low | Low | Low | Low | Low | **Moderate** |
| (Grossmann M 2015) | Cross-sectional | Moderate | Low | Low | Low | Low | Low | Low | **Moderate** |
| (Lin CT et al. 2016) | Cross-sectional | Low | Low | Low | Low | Low | Low | Low | **Low** |
| (Stoumpos S et al. 2018) | Cross-sectional | Moderate | Low | Low | Low | Low | Low | Low | **Moderate** |
| (Fayed A et al. 2019) | Cross-sectional | Low | Low | Low | Low | Low | Low | Low | **Low** |
| (Fathalla, M. et al. 2021) | Cross-sectional | Moderate | Low | Low | Low | Low | Low | Low | **Moderate** |

**Supplemental Table 6 (Stable6)**. GRADE assessment for the HD vs. CT group of this meta-analysis.

| **Author(s):** Kailu Zhang  **Question:** Hormones of hemodialysis woman compared to hormones of healthy control for chronic renal failure women  **Setting:** hormone status of chronic renal failure women  **Bibliography:** | | | | | | | | | | | | |
| --- | --- | --- | --- | --- | --- | --- | --- | --- | --- | --- | --- | --- |
| **Certainty assessment** | | | | | | | **№ of patients** | | **Effect** | | **Certainty** | **Importance** |
| **№ of studies** | **Study design** | **Risk of bias** | **Inconsistency** | **Indirectness** | **Imprecision** | **Other considerations** | **hormones of hemodialysis woman** | **hormones of healthy control** | **Relative (95% CI)** | **Absolute (95% CI)** |  |  |
| **Prolactin (follow-up: range 2 months to 264 months; assessed with: blood testing)** | | | | | | | | | | | | |
| 13 | observational studies | not serious | not serious^a^ | not serious | not serious | strong association | 396 | 366 | - | MD **1.24 SD higher** (0.79 higher to 1.69 higher) | ⨁⨁⨁◯ Moderate | CRITICAL |
| **follicle stimulating hormone (follow-up: range 2 months to 264 months; assessed with: blood testing)** | | | | | | | | | | | | |
| 14 | observational studies | not serious^b^ | not serious^c^ | not serious | not serious | strong association | 408 | 416 | - | SMD **0.89 SD higher** (0.35 higher to 1.44 higher) | ⨁⨁⨁◯ Moderate | IMPORTANT |
| **luteinizing hormone (assessed with: blood testing)** | | | | | | | | | | | | |
| 13 | observational studies | not serious^b^ | not serious^d^ | not serious | not serious | strong association | 370 | 379 | - | SMD **1.39 SD higher** (0.85 higher to 1.93 higher) | ⨁⨁⨁◯ Moderate | IMPORTANT |
| **progestin (follow-up: range 2 months to 264 months; assessed with: blood testing)** | | | | | | | | | | | | |
| 5 | observational studies | not serious^b^ | not serious | not serious | not serious | strong association | 126 | 106 | - | SMD **1.62 SD lower** (2.04 lower to 1.2 lower) | ⨁⨁⨁◯ Moderate | IMPORTANT |
| **estrogen (assessed with: blood testing)** | | | | | | | | | | | | |
| 14 | observational studies | not serious^b^ | not serious^e^ | not serious | not serious | strong association | 379 | 387 | - | SMD **0.4 SD lower** (0.91 lower to 0.12 higher) | ⨁⨁⨁◯ Moderate | IMPORTANT |

**CI:** confidence interval; **MD:** mean difference; **SMD:** standardised mean difference

#### Explanations

a. despite the high heterogeneity, almost all studies showed that the prolactin value was higher in hemodialysis women, which was the purpose of our study

b. most bias came from unclear sampling time in some studies, so we did the subgroup analysis to downgrade the bias

c. the inconsistency was moderate with I2=50% in the subgroup of sampling during follicular phase. When excluding one study with high heterogeneity, the I2=0%. And both before and after excluding the study aforementioned, the overall effect showed the same result that the FSH was higher in hemodialysis women.

d. the inconsistency was high with I2=73% in the subgroup of sampling during follicular phase. When excluding one study with high heterogeneity, the I2=39%. And both before and after excluding the study aforementioned, the overall effect showed the same result that the LH value was higher in hemodialysis women.

e. the inconsistency was higher in subgroup 2 with unclear sampling time. While there was good homogeneity in subgroup 1 which took samples during follicular phase. We drew conclusions largely depending on the subgroup 1, so we thought the inconsistency was not serious in this part.

**Author(s):** Kailu Zhang

**Question:** Hormones of hemodialysis women compared to hormones of renal transplantation for chronic renal failure women

**Setting:** hormone status of chronic renal failure women

**Bibliography:**

| **Certainty assessment** | | | | | | | **№ of patients** | | **Effect** | | **Certainty** | **Importance** |
| --- | --- | --- | --- | --- | --- | --- | --- | --- | --- | --- | --- | --- |
| **№ of studies** | **Study design** | **Risk of bias** | **Inconsistency** | **Indirectness** | **Imprecision** | **Other considerations** | **hormones of hemodialysis women** | **hormones of renal transplantation** | **Relative (95% CI)** | **Absolute (95% CI)** |  |  |
| **Prolactin (follow-up: range 1.8 months to 78 months; assessed with: blood testing)** | | | | | | | | | | | | |
| 6 | observational studies | not serious | not serious^a^ | not serious | not serious | strong association | 121 | 115 | - | MD **0.83 SD higher** (0.27 higher to 1.39 higher) | ⨁⨁⨁◯ Moderate | CRITICAL |
| **follicle-stimulating hormone (follow-up: range 1.8 months to 78 months; assessed with: blood testing)** | | | | | | | | | | | | |
| 6 | observational studies | not serious^b^ | not serious^c^ | not serious | not serious | strong association | 167 | 151 | - | SMD **0.49 SD higher** (0.1 higher to 0.89 higher) | ⨁⨁⨁◯ Moderate | IMPORTANT |
| **luteinizing hormone (follow-up: range 1.8 months to 78 months; assessed with: blood testing)** | | | | | | | | | | | | |
| 6 | observational studies | not serious^b^ | not serious^c^ | not serious | not serious | strong association | 167 | 133 | - | SMD **0.64 SD higher** (0.22 higher to 1.06 higher) | ⨁⨁⨁◯ Moderate | IMPORTANT |
| **Estradiol (follow-up: range 1.8 months to 78 months; assessed with: blood testing)** | | | | | | | | | | | | |
| 6 | observational studies | not serious^d^ | serious^e^ | not serious | not serious | strong association | 162 | 141 | - | SMD **0.47 SD lower** (1.14 lower to 0.2 higher) | ⨁⨁◯◯ Low | IMPORTANT |
| **progesterone (follow-up: range 1.8 months to 78 months; assessed with: blood testing)** | | | | | | | | | | | | |
| 3 | observational studies | not serious | not serious^f^ | not serious | not serious | none | 84 | 66 | - | SMD **0.31 SD lower** (1.32 lower to 0.7 higher) | ⨁⨁◯◯ Low | IMPORTANT |

**CI:** confidence interval; **MD:** mean difference; **SMD:** standardised mean difference

#### Explanations

a. there was higher I2 firstly in this part, and when the study with high heterogeneity was excluded, the I2 lowered from 80% to 0%. And both before and after exclusion, there was the same result that the prolactin value was higher in hemodialysis women.

b. the bias mainly came from different sampling time, so we did the subgroup analysis and draw conclusions depended on those studies taking samles during follicular phase.

c. there was lower I2 in the subgroup taking samples during follicular phase

d. there was unclear selecion bias, no measuring bias and reporting bias. but selecting of patients had little to do with the hormones outcome if samping time and age were considered. In this comparison, we did subgroup analysis to control bias. Age was compariable between hemodialysis women and control in every study.

e. there was higher I2 in this comparison.

f. There was higher I2 in this comparison, but data in the study of Stoumpos S et al. were significantly skewed away from normality and it is thus not recommended to apply the normal-based methods for data transformation. When this study was excluded, there was large homogeneity.
